# Counterexamples to regularities for the derivative processes associated to stochastic evolution equations

**DOI:** 10.1007/s40072-024-00342-z

**Published:** 2025-02-04

**Authors:** Mario Hefter, Arnulf Jentzen, Ryan Kurniawan

**Affiliations:** 1 Department of Mathematics, University of Kaiserslautern-Landau (RPTU), Kaiserslautern, Germany; 2 School of Data Science & Shenzhen Research Institute of Big Data, The Chinese University of Hong Kong, Shenzhen (CUHK-Shenzhen), Shenzhen, China; 3 Applied Mathematics: Institute for Analysis and Numerics, Faculty of Mathematics and Computer Science, University of Münster, Münster, Germany; 4 Seminar for Applied Mathematics, Department of Mathematics, ETH Zurich, Zurich Switzerland

## Abstract

In the recent years there has been an increased interest in studying regularity properties of the derivatives of semilinear parabolic stochastic evolution equations (SEEs) with respect to their initial values. In particular, in the scientific literature it has been shown for every natural number $n\in {\mathbb {N}}$ that if the nonlinear drift coefficient and the nonlinear diffusion coefficient of the considered SEE are *n*-times continuously Fréchet differentiable, then the solution of the considered SEE is also *n*-times continuously Fréchet differentiable with respect to its initial value and the corresponding derivative processes satisfy a suitable regularity property in the sense that the *n*-th derivative process can be extended continuously to *n*-linear operators on negative Sobolev-type spaces with regularity parameters $\delta _1,\delta _2,\ldots ,\delta _n\in [0,\infty )$ provided that the condition $\sum ^n_{i=1} \delta _i < \nicefrac {1}{2}$ is satisfied. The main contribution of this paper is to reveal that this condition can essentially not be relaxed.

## Introduction

In the recent years there has been an increased interest in studying regularity properties of the derivatives of stochastic evolution equations (SEEs) with respect to their initial values (cf., e.g., Cerrai [8, Chapters 6–7], Da Prato & Zabczyk [11, Theorem 7.3.6], Debussche [12, Lemmas 4.4-$-$4.6], Wang & Gan [17, Lemma 3.3], and Andersson et al. [2, 3]). One important reason for this increased interest is that appropriate estimates on the first, second, and higher order derivatives of SEEs with respect to their initial values have been used as key tools for establishing essentially sharp weak convergence rates; see, e.g., Debussche [12, Theorem 2.2], Wang & Gan [17, Theorem 2.1], Andersson & Larsson [4, Theorem 1.1], Bréhier [5, Theorem 1.1], Bréhier & Kopec [7, Theorem 5.1], Wang [16, Corollary 1], Conus et al. [9, Corollary 5.2], [14, Corollary 8.2], and Hefter et al. [13, Theorem 1.1]. In particular, the article Conus et al. [9] establishes for the first time weak convergence rates for SPDEs with nonlinear diffusion coefficients (a) by applying the standard Itô formula to the difference between the exact solution process and a certain modified version of its numerical approximation process (see (9) in Conus et al. [9]), (b) by employing the infinite dimensional Kolmogorov backward PDE associated to the SPDE under consideration, and (c) by applying the mild Itô formula in Da Prato et al. [10] to the error terms resulting from (a) and (b). In this procedure one needs appropriate bounds for the first four derivatives of the solution of the infinite dimensional Kolmogorov backward PDE associated to the SEE under consideration (the first and the second spatial derivatives appear according to (a) and (b) and the third and fourth spatial derivatives appear due to applications of the mild Itô formula in Da Prato et al. [10] according to (c) above). The Feynman-Kac formula tells us that the solution of the Kolmogorov backward PDE is nothing else but the expectation of the test function evaluated at the solution process of the associated SEE. The first four spatial derivatives of the solution of the Kolmogorov backward PDE are thus strongly linked to the first four derivatives with respect to the initial value of the solution process of the SEE under consideration. This illustrates the reason why suitable a priori bounds for derivatives with respect to the initial value of solutions of SEEs (see item (iii) in Theorem 1.1 below) are key tools for establishing sharp weak convergence rates as in Conus et al. [9]. The fundamental importance of suitable a priori bounds for derivatives of solutions of SEEs with respect to their initial values is one important reason why in recent years there has been an increased interest in studying the regularity properties for derivatives of SEEs with respect to their initial values. In particular, in the recent article Andersson et al. [3] it has been shown that if the nonlinear drift coefficient and the nonlinear diffusion coefficient of an SEE are *n*-times continuously Fréchet differentiable, then the solution of the considered SEE is also *n*-times continuously Fréchet differentiable with respect to its initial value and the corresponding derivative processes satisfy a suitable regularity property (see item (iv) of Theorem 1.1 in Andersson et al. [3] and item (iii) of Theorem 1.1 below, respectively). In this work we reveal that this regularity property can essentially not be improved. To illustrate our result in more detail we consider the following notation throughout the rest of this introductory section. For every measure space , every measurable space , and every /-measurable function $X :\Omega \rightarrow S$ we denote by  the set given by1We first briefly review the above mentioned regularity result on derivative processes of SEEs from Andersson et al. [3]. More formally, Theorem 1.1 in Andersson et al. [3] immediately implies the following result.

### Theorem 1.1

(see Theorem 1.1 in Andersson et al. [3]) For every real number $T\in (0,\infty )$, all nontrivial separable ${\mathbb {R}}$-Hilbert spaces $( H, \left\| \cdot \right\| _H, \langle \cdot , \cdot \rangle _H )$ and $( U, \left\| \cdot \right\| _U, \langle \cdot , \cdot \rangle _U )$, every probability space , every normal filtration  on , every $\operatorname {Id}_U$-cylindrical -Wiener process $(W_t)_{t\in [0,T]}$, every generator $A:D(A)\subseteq H \rightarrow H$ of a strongly continuous analytic semigroup with $\operatorname {spectrum}(A) \subseteq \{z\in \mathbb {C}:\operatorname {Re}(z)<0\}$, and all infinitely often Fréchet differentiable functions $F :H \rightarrow H$ and $B :H \rightarrow HS(U,H)$ with globally bounded derivatives we have

(i) that there exist up-to-modifications unique /-predictable stochastic processes $X^x :[0,T] \times \Omega \rightarrow H$, $x \in H$, such that for all $x \in H$, $p \in [2,\infty )$, $t \in [0,T]$ it holds that $\int ^t_0 \Vert e^{(t-s)A} F(X^x_s)\Vert _H + \Vert e^{(t-s)A} B(X^x_s)\Vert ^2_{HS(U,H)} \, ds < \infty$, $\sup _{ s \in [0,T] } {\mathbb {E}}\big [\Vert X_s^x \Vert ^p_H \big ] < \infty$, and 2
(ii) that it holds for all $p \in [2,\infty )$, $t \in [0,T]$ that  is infinitely often Fréchet differentiable, and
(iii) that it holds for all $p \in [2,\infty )$, $n \in {\mathbb {N}}= \{1,2,\ldots \}$, $q\in [0,\infty )$, $\delta _1, \delta _2, \ldots , \delta _n \in [0,\nicefrac {1}{2})$, $t \in (0,T]$ with $\sum ^n_{i=1} \delta _i < \nicefrac {1}{2}$ that 3

Item (iv) of Theorem 1.1 in Andersson et al. [3] and item (iii) of Theorem 1.1 in this paper, respectively, prove that the condition

4 $$\begin{aligned} \sum ^n_{i=1} \delta _i < \nicefrac {1}{2} \end{aligned}$$

for the regularity parameters $\delta _1,\delta _2,\ldots ,\delta _n \in [0,\nicefrac {1}{2})$ of the considered negative Sobolev-type spaces is sufficient to ensure that the left-hand side of (3) is finite. The main result of this work (see Theorem 1.2 below and Theorem 2.1 in Sect. 2.5 below, respectively) reveals that this condition can essentially not be relaxed. More specifically, Theorem 2.1 in Sect. 2.5 below directly implies the following result.

### Theorem 1.2

(see Theorem 2.1 in Sect. 2.5 of this article below) For every real number $T\in (0,\infty )$, every infinite dimensional separable ${\mathbb {R}}$-Hilbert space $( H, \left\| \cdot \right\| _H, \langle \cdot , \cdot \rangle _H )$, every nontrivial separable ${\mathbb {R}}$-Hilbert space $( U, \left\| \cdot \right\| _U, \langle \cdot , \cdot \rangle _U )$, every probability space , every normal filtration  on , and every $\operatorname {Id}_U$-cylindrical -Wiener process $(W_t)_{t\in [0,T]}$ there exist a generator $A:D(A)\subseteq H \rightarrow H$ of a strongly continuous analytic semigroup with $\operatorname {spectrum}(A) \subseteq \{z\in \mathbb {C}:\operatorname {Re}(z)<0\}$ and infinitely often Fréchet differentiable functions $F :H \rightarrow H$ and $B :H \rightarrow HS(U,H)$ with globally bounded derivatives such

(i) that there exist up-to-modifications unique /-predictable stochastic processes $$ X^x :$$ $$ [0,T] \times \Omega \rightarrow H $$, $$ x \in H $$, such that for all $$ x \in H $$, $$ p \in [2,\infty ) $$, $$ t \in [0,T] $$ it holds that $$ \int ^t_0 \Vert e^{(t-s)A} F(X^x_s)\Vert _H + \Vert e^{(t-s)A} B(X^x_s)\Vert ^2_{HS(U,H)} \, ds < \infty $$, $$ \sup _{ s \in [0,T] } {\mathbb {E}}\big [\Vert X_s^x \Vert ^p_H \big ] < \infty $$, and 5
(ii) that it holds for all $p \in [2,\infty )$, $t \in [0,T]$ that  is infinitely often Fréchet differentiable,
(iii) that it holds for all $p \in [2,\infty )$, $n \in {\mathbb {N}}$, $q\in [0,\infty )$, $\delta _1, \delta _2, \ldots , \delta _n \in [0,\nicefrac {1}{2})$, $t \in (0,T]$ with $\sum ^n_{i=1} \delta _i < \nicefrac {1}{2}$ that 6 and
(iv) that it holds for all $p \in [2,\infty )$, $n \in {\mathbb {N}}$, $q\in [0,\infty )$, $\delta _1, \delta _2, \ldots , \delta _n \in {\mathbb {R}}$, $t \in (0,T]$ with $\sum ^n_{i=1} \delta _i > \nicefrac {1}{2}$ that 7

Item (iii) in Theorem 1.2 above also reveals that the hypothesis that $\sum ^n_{i=1} \delta _i < \nicefrac {1}{2}$ is essentially optimal. Clearly, there are cases where the suprema in (6) above is finite even though the condition that $\sum ^n_{i=1} \delta _i < \nicefrac {1}{2}$ is not satisfied (cf., e.g., Debussche [12, Lemmas 4.4-$-$4.6] and Andersson et al. [2, Corollary 2.10]), for instance, the case where *B* vanishes, the case where *B* is a constant function (cf., e.g., Wang & Gan [17, Lemma 3.3]), and the case where *B* is a linear function (cf., e.g., Andersson et al. [2, Proposition 3.3]). Nonetheless, the main contribution of Theorem 1.2 is to reveal that under the general assumptions in Theorem 1.1 it holds that $\sum ^n_{i=1} \delta _i < \nicefrac {1}{2}$ is essentially optimal. Regularity results for Kolmogorov equations associated to SEEs of the form (2) and (5), which are in some sense related to Corollaries 1.1 and 1.2, can, e.g., be found in Debussche [12, Lemmas 4.4-$-$4.6], Wang & Gan [17, Lemma 3.3], Andersson & Larsson [4, (4.2)–(4.3)], Bréhier [5, Propositions 5.1-$-$5.2 and Lemma 5.4], Wang [16, Lemma 3.3], Andersson et al. [1, Theorem 3.3], and Brehier & Debussche [6, Theorems 3.2-$-$3.3 and Proposition 3.5].

The remainder of this article is organized as follows. In Sect. 2 we state and prove the main result of this paper; see Theorem 2.1 in Sect. 2.5 below. In Sect. 2.1 we present the drift and the diffusion coefficient functions that we use throughout Sect. 2. In Sect. 2.2 we outline the proof of the main result of this article; see Theorem 1.2 above and Theorem 2.1 below respectively. In Sect. 2.3 we derive an explicit representation of the considered diffusion coefficient function (see Lemma 2.1 in Sect. 2.3). In Sect. 2.4 we present explicit formulas for the solution and its derivatives of the SEE associated with the drift and diffusion coefficient functions considered in Sect. 2.1 (see Lemma 2.2 in Sect. 2.4). In Sect. 2.5 we employ Lemma 2.1 in Sect. 2.3 and Lemma 2.2 in Sect. 2.4 to prove the main result of this paper, Theorem 2.1 in Sect. 2.5. Theorem 1.2 above is an immediate consequence of Theorem 2.1 in Sect. 2.5.

## Counterexamples to regularities for the derivative processes associated to stochastic evolution equations

### Setting

Throughout this section we consider the following setting. For every set *A* let  be the power set of *A* and let $\#_A \in {\mathbb {N}}_0 \cup \{\infty \}$ be the number of elements of *A*, let , $k \in {\mathbb {N}}_0$, satisfy for all $k \in {\mathbb {N}}$ that $\Pi _0=\emptyset$ and8(the set of partitions of $\{1,2,\ldots ,k\}$; see, e.g., (10) in Andersson et al. [3]), let $( H, \left\| \cdot \right\| _H, \langle \cdot , \cdot \rangle _H )$ be an ${\mathbb {R}}$-Hilbert space, let $e = ( e_n )_{ n \in {\mathbb {N}}} :{\mathbb {N}}\rightarrow H$ be an orthonormal basis of *H*, let $\lambda = ( \lambda _n )_{ n \in {\mathbb {N}}} :{\mathbb {N}}\rightarrow {\mathbb {R}}$, $P :H \rightarrow H$, and $B :H \rightarrow H$ satisfy for all $v \in H$ that

9 $$\begin{aligned} \sup _{ n \in {\mathbb {N}}} \lambda _n < 0, \qquad P v = \sum _{ n = 2 }^{ \infty } \langle e_n, v \rangle _H e_n, \qquad \text {and}\qquad B( v ) = \sqrt{ 1 + \Vert P v \Vert ^2_H } \, e_1, \end{aligned}$$

let $T \in (0,\infty )$, let $( \Omega , \mathcal {F}, {\mathbb {P}})$ be a probability space with a normal filtration $(\mathcal {F}_t)_{t\in [0,T]}$, let $W :[0,T] \times \Omega \rightarrow {\mathbb {R}}$ be a standard $( \Omega , \mathcal {F}, {\mathbb {P}}, ( \mathcal {F}_t )_{ t \in [0,T] } )$-Brownian motion, let $A :D(A) \subseteq H \rightarrow H$ satisfy $D(A) = \{ v \in H :\sum ^\infty _{n=1} \left| \lambda _n \langle e_n,v \rangle _H \right| ^2 < \infty \}$ and $\forall \, v \in D(A) :Av = \sum ^\infty _{n=1} \lambda _n \langle e_n,v\rangle _H e_n$, let $( H_r, \left\| \cdot \right\| _{ H_r }, \langle \cdot , \cdot \rangle _{ H_r } )$, $r \in {\mathbb {R}}$, be a family of interpolation spaces associated to $- A$ (cf., e.g., [15, Section 3.7]), and for every /-measurable function $X :\Omega \rightarrow H$ let $|\![X]\!|$ be the set given by $|\![X]\!| = \big \{ Y :\Omega \rightarrow H :( Y \text { is }\mathcal {F}\text {/}\mathcal {B}(H)\text {-}$ $\text {-measurable and } {\mathbb {P}}(X=Y) = 1 ) \big \}$.

### Outline of the proof of the main result of this article

In this paragraph we briefly sketch some of the arguments in our proof of Theorem 1.2 above and Theorem 2.1 below, respectively. We first show in Lemma 2.1 below that *B* is infinitely often Fréchet differentiable with globally bounded derivatives. This together with Andersson et al. [3, Theorem 2.1] establishes items (i)–(iv) of Theorem 2.1 below (see also items (i)–(iv) of Lemma 2.2 below). It thus remains to sketch our proof strategy for item (v) of Theorem 2.1. In the following we outline the two key ingredients to prove this item. The first key ingredient is an explicit representation formula for the derivatives of the diffusion coefficient *B*. To be more specific, item (ii) of Lemma 2.1 below shows that for all $m,n\in {\mathbb {N}}$, $t\in (0,T]$, $u_{0,m},u_{1,m},\ldots ,u_{n,m}\in {(\cap _{r\in {\mathbb {R}}}H_r)}\setminus \{0\}$ it holds that

10 $$\begin{aligned} \begin{aligned}&B^{ ( n ) }( e^{tA} u_{0,m} ) \big ( e^{tA} u_{1,m} , e^{tA} u_{2,m} , \dots , e^{tA} u_{n,m} \big ) \\&= \Bigg ( {\sum \limits _{ \varpi \in \Pi _n }} \frac{ \big [ {\prod ^{ \#_\varpi - 1 }_{ i=0 }} (1-2i) \big ] \big [ \prod \nolimits _{ I \in \varpi } \langle \mathbb {1}_{\{1,2\}}( \#_I ) \, Pe^{tA} u_{\max (I) {\mathbb {1}}_{\{2\}}(\#_I),m} , e^{tA} u_{ \min (I),m } \rangle _H \big ] }{ \big [ 1 + \Vert Pe^{tA} u_{0,m} \Vert ^2_H \big ]^{ ( \#_\varpi - \nicefrac {1}{2} ) } } \Bigg ) \, e_1 . \end{aligned} \end{aligned}$$

The second key ingredient is a suitable explicit representation formula for the derivatives of the solution process of the SEE

11 $$\begin{aligned} dX_t = AX_t \, dt + B(X_t) \, dW_t \end{aligned}$$

with respect to its initial value. To be more specific, item (v) of Lemma 2.2 below shows that for all $q\in [0,\infty )$, $m,n\in {\mathbb {N}}$, $t\in (0,T]$ and all $\textbf{u}_l = (u_{0,l},u_{1,l},\ldots ,u_{n,l}) \in ({(\cap _{r\in {\mathbb {R}}}H_r)}\setminus \{0\})^{n+1}$, $l\in {\mathbb {N}}$, it holds that

12 $$\begin{aligned} |\![X_t^{n,\textbf{u}_m} - \mathbb {1}_{ \{ 0, 1 \} }(n) \, e^{tA} u_{n,m} ]\!| = \int _0^t e^{ ( t - s ) A } B^{ ( n ) }( e^{sA} u_{0,m} ) \big ( e^{sA} u_{1,m} , e^{sA} u_{2,m} , \dots , e^{sA} u_{n,m} \big ) \, dW_s . \end{aligned}$$

With these two key ingredients in hand, the first step of our proof strategy for item (v) of Theorem  2.1 is to note that for all $n\in {\mathbb {N}}$, $q\in [0,\infty )$, $p\in [2,\infty )$, $t\in (0,T]$ and every sequence of vectors $(\textbf{u}_m)_{m\in {\mathbb {N}}} = (u_{0,m},u_{1,m},\ldots ,u_{n,m})_{m\in {\mathbb {N}}} \subseteq ({(\cap _{r\in {\mathbb {R}}}H_r)}\setminus \{0\})^{n+1}$ it holds that

13 $$\begin{aligned} \begin{aligned}&\sup _{ \textbf{u}=(u_0,u_1,\ldots ,u_n) \in ({(\cap _{r\in {\mathbb {R}}}H_r)}\setminus \{0\})^{n+1} } \frac{ \big ({\mathbb {E}}\big [ \Vert X^{ n,\textbf{u} }_t \Vert ^p_{H_{-q}} \big ]\big )^{\nicefrac {1}{p}} }{ \prod ^{ n }_{ i=1 } \Vert u_i\Vert _{H_{-\delta _i}} } \\  &\ge \sup _{ m\in {\mathbb {N}}} \frac{ \big ({\mathbb {E}}\big [ \Vert X^{ n,\textbf{u}_m }_t \Vert ^p_{H_{-q}} \big ]\big )^{\nicefrac {1}{p}} }{ \prod ^{ n }_{ i=1 } \Vert u_{i,m}\Vert _{H_{-\delta _i}} } \\  &\ge \left[ \inf _{ m\in {\mathbb {N}}} \frac{1}{ \prod ^n_{i=1} \Vert u_{i,m} \Vert _{H_{-\delta _i}} } \right] \left[ \sup _{ m\in {\mathbb {N}}} \big ({\mathbb {E}}\big [ \Vert X^{ n,\textbf{u}_m }_t \Vert ^p_{H_{-q}} \big ]\big )^{\nicefrac {1}{p}} \right] . \end{aligned} \end{aligned}$$

This and Hölder’s inequality imply that for all $n\in {\mathbb {N}}$, $q\in [0,\infty )$, $p\in [2,\infty )$, $t\in (0,T]$ and every sequence of vectors $(\textbf{u}_m)_{m\in {\mathbb {N}}} = (u_{0,m},u_{1,m},\ldots ,u_{n,m})_{m\in {\mathbb {N}}} \subseteq ({(\cap _{r\in {\mathbb {R}}}H_r)}\setminus \{0\})^{n+1}$ it holds that

14 $$\begin{aligned} \begin{aligned}&\sup _{ \textbf{u}=(u_0,u_1,\ldots ,u_n) \in ({(\cap _{r\in {\mathbb {R}}}H_r)}\setminus \{0\})^{n+1} } \frac{ \big ({\mathbb {E}}\big [ \Vert X^{ n,\textbf{u} }_t \Vert ^p_{H_{-q}} \big ]\big )^{\nicefrac {1}{p}} }{ \prod ^{ n }_{ i=1 } \Vert u_i\Vert _{H_{-\delta _i}} } \\  &\ge \left[ \inf _{ m\in {\mathbb {N}}} \frac{1}{ \prod ^n_{i=1} \Vert u_{i,m} \Vert _{H_{-\delta _i}} } \right] \left[ \sup _{ m\in {\mathbb {N}}} \big ({\mathbb {E}}\big [ \Vert X^{ n,\textbf{u}_m }_t \Vert ^2_{H_{-q}} \big ]\big )^{\nicefrac {1}{2}} \right] . \end{aligned} \end{aligned}$$

Taking this inequality into account, the second step of our proof strategy for item (v) of Theorem 2.1 is to find for every $n\in {\mathbb {N}}$, $q\in [0,\infty )$ a sequence of vectors $(\textbf{u}_m)_{m\in {\mathbb {N}}} \subseteq ({(\cap _{r\in {\mathbb {R}}}H_r)}\setminus \{0\})^{n+1}$ such that for all $t\in (0,T]$ it holds that

15 $$\begin{aligned} \sup _{ m\in {\mathbb {N}}} \prod ^n_{i=1} \Vert u_{i,m} \Vert _{H_{-\delta _i}} < \infty \qquad \text {and}\qquad \sup _{ m\in {\mathbb {N}}} \big ({\mathbb {E}}\big [ \Vert X^{ n,\textbf{u}_m }_t \Vert ^2_{H_{-q}} \big ]\big )^{\nicefrac {1}{2}} = \infty . \end{aligned}$$

To narrow our search we use the key ingredients. In particular, we consider the integrand of the stochastic integral in (12) above and we consider the representation for this integrand based on (10) above. We note that the terms of summation in (10) can be greatly reduced to a single term by utilizing the inner products in the numerator of the right-hand side of (10). To be more specific, in the proof of Theorem 2.1 below for all $m,n\in {\mathbb {N}}$ we select the sets $\{u_{0,m},u_{1,m},u_{2,m},\ldots ,u_{2n-1,m}\} \subseteq ({(\cap _{r\in {\mathbb {R}}}H_r)}\setminus \{0\})^{2n}$ and $\{v_{0,m},v_{1,m},v_{2,m},\ldots ,v_{2n,m}\} \subseteq ({(\cap _{r\in {\mathbb {R}}}H_r)}\setminus \{0\})^{2n+1}$ such that for all $\varpi \in \Pi _{2n-1}{\setminus }\{\{ \{1,2\}, \{3,4\}, \ldots , \{2n-3,2n-2\}, \{2n-1\} \}\}$, $\tilde{\varpi }\in \Pi _{2n}{\setminus }\{\{ \{1,2\}, \{3,4\}, \ldots , \{2n-3,2n-2\}, \{2n-1,2n\} \}\}$, $t\in (0,T]$ it holds that

16 $$\begin{aligned} \begin{aligned} \prod \nolimits _{ I \in \varpi } \langle \mathbb {1}_{\{1,2\}}( \#_I ) \, Pe^{tA} u_{\max (I) {\mathbb {1}}_{\{2\}}(\#_I),m} , e^{tA} u_{ \min (I),m } \rangle _H&=0,\\ \prod \nolimits _{ I \in \tilde{\varpi } } \langle \mathbb {1}_{\{1,2\}}( \#_I ) \, Pe^{tA} v_{\max (I) {\mathbb {1}}_{\{2\}}(\#_I),m} , e^{tA} v_{ \min (I),m } \rangle _H&=0. \end{aligned} \end{aligned}$$

Indeed, formula (10) implies that for all $m,n\in {\mathbb {N}}$, $t\in (0,T]$ and all sets $\{u_{0,m},u_{1,m},\ldots ,u_{2n-1,m}\} \subseteq ({(\cap _{r\in {\mathbb {R}}}H_r)}\setminus \{0\})^{2n}$ and $\{v_{0,m},v_{1,m},\ldots ,v_{2n,m}\} \subseteq ({(\cap _{r\in {\mathbb {R}}}H_r)}\setminus \{0\})^{2n+1}$ satisfying (16) it holds that

17 $$\begin{aligned} \begin{aligned}&B^{ ( 2n-1 ) }( e^{tA} u_{0,m} ) \big ( e^{tA} u_{1,m} , e^{tA} u_{2,m} , \dots , e^{tA} u_{2n-1,m} \big ) \\  &= \Bigg ( \sum _{ \varpi \in \Pi _{2n-1}, \, \varpi = \{ \{1,2\}, \{3,4\}, \ldots , \{2n-3,2n-2\}, \{2n-1\} \} } \frac{ \big [ {\prod ^{ \#_\varpi - 1 }_{ i=0 }} (1-2i) \big ] }{ \big [ 1 + \Vert Pe^{tA} u_{0,m} \Vert ^2_H \big ]^{ ( \#_\varpi - \nicefrac {1}{2} ) } } \\  &\quad \cdot \langle Pe^{tA} u_{2n-1,m} , e^{tA} u_{ 2n-1,m } \rangle _H \Bigg [ \prod ^{n-1}_{i=1} \langle Pe^{tA} u_{2i-1,m} , e^{tA} u_{ 2i,m } \rangle _H \Bigg ] \Bigg ) \, e_1 \\  &= \frac{ (1-2i)^{(n-1)} \, \langle Pe^{tA} u_{2n-1,m} , e^{tA} u_{ 2n-1,m } \rangle _H }{ \big [ 1 + \Vert Pe^{tA} u_{0,m} \Vert ^2_H \big ]^{ ( n - \nicefrac {1}{2} ) } } \Bigg [ \prod ^{n-1}_{i=1} \langle Pe^{tA} u_{2i-1,m} , e^{tA} u_{ 2i,m } \rangle _H \Bigg ] \, e_1 \end{aligned} \end{aligned}$$

and

18 $$\begin{aligned} \begin{aligned}&B^{ ( 2n ) }( e^{tA} v_{0,m} ) \big ( e^{tA} v_{1,m} , e^{tA} v_{2,m} , \dots , e^{tA} v_{2n,m} \big ) \\  &= \Bigg ( \sum _{ \tilde{\varpi } \in \Pi _{2n}, \, \tilde{\varpi } = \{ \{1,2\}, \{3,4\}, \ldots , \{2n-3,2n-2\}, \{2n-1,2n\} \} } \frac{ \big [ {\prod ^{ \#_{\tilde{\varpi }} - 1 }_{ i=0 }} (1-2i) \big ] }{ \big [ 1 + \Vert Pe^{tA} u_{0,m} \Vert ^2_H \big ]^{ ( \#_{\tilde{\varpi }} - \nicefrac {1}{2} ) } } \\  &\quad \cdot \Bigg [ \prod ^{n}_{i=1} \langle Pe^{tA} u_{2i-1,m} , e^{tA} u_{ 2i,m } \rangle _H \Bigg ] \Bigg ) \, e_1 \\  &= \frac{ (1-2i)^{(n-1)} }{ \big [ 1 + \Vert Pe^{tA} u_{0,m} \Vert ^2_H \big ]^{ ( n - \nicefrac {1}{2} ) } } \, \Bigg [ \prod ^{n}_{i=1} \langle Pe^{tA} u_{2i-1,m} , e^{tA} u_{ 2i,m } \rangle _H \Bigg ] \, e_1 \end{aligned} \end{aligned}$$

(cf. (83) and (97) below). This motivates us to choose $(u_{0,m},u_{1,m},\ldots ,u_{n,m}) \in ({(\cap _{r\in {\mathbb {R}}}H_r)}\setminus \{0\})^{n+1}$, $m,n\in {\mathbb {N}}$, such that for all $m,n\in {\mathbb {N}}$, $i\in \{1,2,\ldots ,n\}$ it holds that

19 $$\begin{aligned} u_{i,m}&= (-A)^{r_{i,n}} \left[ \sum ^{m}_{ j=1 } K_{i,n,m}\, e_{k_{i,n}+jl_{n,m}} \right] = \sum ^m_{ j=1 } [ c \, (k+ij)^2 ]^r \, e_{k+ij}\nonumber \\  &= c^r \left[ \sum ^m_{j=1} (k+ij)^{2r} \, e_{k+ij} \right] \end{aligned}$$

for some $l_{n,m},K_{i,n,m}\in {\mathbb {N}}$, $i\in \{1,2,\ldots ,n\}$, $m,n\in {\mathbb {N}}$, $k_{i,n}\in {\mathbb {N}}_0$, $i\in \{1,2,\ldots ,n\}$, $n\in {\mathbb {N}}$, $r_{i,n}\in {\mathbb {R}}$, $i\in \{1,2,\ldots ,n\}$, $n\in {\mathbb {N}}$ (cf. (64)–(67) below). Indeed, for all $m,n\in {\mathbb {N}}$, $K_1,k_2\in \{1,2,\ldots ,n\}$, $r_1,r_2\in {\mathbb {R}}$, $t\in [0,T]$ it holds that

20 $$\begin{aligned} \langle P e^{tA} v^{k_1,r_1}_{n,N}, e^{tA} v^{k_2,r_2}_{n,N} \rangle _H = {\mathbb {1}}_{\{k_1\}}(k_2) \, \Vert P e^{tA} v^{k_1,\nicefrac {(r_1+r_2)}{2}}_{n,N} \Vert ^2_H \end{aligned}$$

(see (78) below). This assures that our choice of $(u_{0,m},u_{1,m},\ldots ,u_{n,m}) \in ({(\cap _{r\in {\mathbb {R}}}H_r)}\setminus \{0\})^{n+1}$, $m,n\in {\mathbb {N}}$, in (64)–(67) below satisfies (16). Next we use the second key ingredient (see (12) above) to show that for all $n \in {\mathbb {N}}$, $r\in [0,\infty )$, $\textbf{u}= (u_0, u_1, \ldots , u_n) \in H^{n+1}$, $t\in [0,T]$ it holds that

21 $$\begin{aligned} {\mathbb {E}}\big [ \Vert X^{ n,\textbf{u} }_t \Vert ^2_{H_{-r}} \big ] \ge \int ^t_0 \Vert e^{(t-s)A } B^{(n)}( e^{sA} u_0 ) ( e^{sA} u_1, e^{sA} u_2, \ldots , e^{sA} u_n ) \Vert ^2_{H_{-r}} \,ds \end{aligned}$$

(see (75) below and its direct application in (76)–(77)) and to substitute (17)–(19) into (21) (cf. (92), (100) and (101) below). Finally, we use the assumption that $\forall \, n\in {\mathbb {N}}: \sum ^n_{i=1} \delta _i > \nicefrac {1}{2}$ to determine the right choice of the families of parameters $l_{n,m},K_{i,n,m}\in {\mathbb {N}}$, $i\in \{1,2,\ldots ,n\}$, $m,n\in {\mathbb {N}}$, $k_{i,n}\in {\mathbb {N}}_0$, $i\in \{1,2,\ldots ,n\}$, $n\in {\mathbb {N}}$, $r_{i,n}\in {\mathbb {R}}$, $i\in \{1,2,\ldots ,n\}$, $n\in {\mathbb {N}}$, for (19) such that for all $n\in {\mathbb {N}}$, $q\in [0,\infty )$, $t\in (0,T]$ it holds that there exists a positive real number $C\in (0,\infty )$ such that

22 $$\begin{aligned} \sup _{ m\in {\mathbb {N}}} {\mathbb {E}}\big [ \Vert X^{ n,\textbf{u}_m }_t \Vert ^2_{H_{-q}} \big ] \ge C\cdot \sup _{m\in {\mathbb {N}}}\left[ \sum ^m_{j,k=1}\frac{1}{(j^2+k^2)}\right] = \infty \end{aligned}$$

(cf. (106) in the proof of Theorem 2.1 below).

### An explicit representation for the diffusion coefficient

#### Lemma 2.1

(Derivatives of the diffusion *B*) Assume the setting in Sect. 2.1. Then

(i) it holds that $B :H \rightarrow H$ is infinitely often differentiable,
(ii) it holds for all $n \in {\mathbb {N}}$, $v_0, v_1, \ldots , v_n \in H$ that
  23 $$\begin{aligned} \begin{aligned}&B^{(n)}( v_0 )( v_1,v_2,\ldots ,v_n ) \\  &= \Bigg ( {\sum \limits _{ \varpi \in \Pi _n }} \frac{ \big [ {\prod ^{ \#_\varpi - 1 }_{ i=0 }} (1-2i) \big ] \big [ \prod \nolimits _{ I \in \varpi } \langle \mathbb {1}_{\{1,2\}}( \#_I ) \, Pv_{\max (I) {\mathbb {1}}_{\{2\}}(\#_I)} , v_{ \min (I) } \rangle _H \big ] }{ \big [ 1 + \Vert Pv_0 \Vert ^2_H \big ]^{ ( \#_\varpi - \nicefrac {1}{2} ) } } \Bigg ) \, e_1 , \end{aligned} \end{aligned}$$
  and
(iii) it holds for all $n \in {\mathbb {N}}$ that $\sup _{v\in H} \Vert B^{(n)}(v)\Vert _{L^{(n)}(H,H)} < \infty$.

#### Proof

Throughout this proof let $f \in C^\infty ( (0,\infty ), {\mathbb {R}})$ and $g \in C^\infty ( H, (0,\infty ) )$ satisfy for all $x \in (0,\infty )$, $v \in H$ that

24 $$\begin{aligned} f(x) = \sqrt{x} \qquad \text {and}\qquad g(v) = 1+\Vert Pv \Vert ^2_H \end{aligned}$$

and let $I^\varpi _i \in \varpi$, $i \in \{1,2,\ldots ,\#_\varpi \}$, $\varpi \in \Pi _n$, $n \in {\mathbb {N}}$, satisfy for all $n \in {\mathbb {N}}$, $\varpi \in \Pi _n$ that

25 $$\begin{aligned} \min ( I^\varpi _1 )< \min ( I_2^\varpi )< \dots < \min ( I_{ \#_\varpi }^{ \varpi } ) . \end{aligned}$$

Note that the fact that $\forall \, v \in H :B(v) = (f\circ g)(v) \, e_1 = f(g(v))\, e_1$ proves item (i).

In the next step we prove (23) by induction on $n\in {\mathbb {N}}$. For the base case $n=1$ we note that for all $v_0, v_1 \in H$ it holds that

26 $$\begin{aligned} \begin{aligned} B'(v_0) v_1&= \big [ (f \circ g)'(v_0) v_1 \big ] e_1 = \big [ (f' \circ g)(v_0) g'(v_0) v_1 \big ] e_1 \\  &= \frac{1}{ [1+\Vert P v_0\Vert ^2_H]^{\nicefrac {1}{2}} } \langle P v_0, P v_1 \rangle _H \, e_1 = \frac{ \langle P v_0, v_1 \rangle _H }{ [1+\Vert P v_0\Vert ^2_H]^{\nicefrac {1}{2}} }\, e_1 . \end{aligned} \end{aligned}$$

This and the fact that $\Pi _1 = \{\{\{1\}\}\}$ prove (23) in the base case $n=1$. For the induction step ${\mathbb {N}}\ni n \rightarrow n+1 \in \{2,3,\ldots \}$ assume that (23) holds for some natural number $n \in {\mathbb {N}}$. Observe that item (i), the induction hypothesis, and the product rule of differentiation ensure that for all $v_0, v_1, \ldots , v_{n+1} \in H$ it holds that

27 $$\begin{aligned} \begin{aligned}&B^{(n+1)}( v_0 )( v_1,v_2,\ldots ,v_{n+1} ) \\  &= \big ( \tfrac{d}{d v_0} \big [ B^{(n)}( v_0 )( v_1,v_2,\ldots ,v_n ) \big ]\big ) v_{n+1} \\  &= \Bigg ( \sum _{ \varpi \in \Pi _n } \left[ {\textstyle \prod ^{ \#_\varpi - 1 }_{ j=0 }} (1-2j) \right] \Bigg ( \frac{d}{d v_0} \Bigg [ \frac{ \prod _{ I \in \varpi } \langle \mathbb {1}_{\{1,2\}}( \#_I ) \, Pv_{\max (I) {\mathbb {1}}_{\{2\}}(\#_I)} , v_{ \min (I) } \rangle _H }{ [ 1 + \Vert Pv_0 \Vert ^2_H ]^{ ( \#_\varpi - \nicefrac {1}{2} ) } } \Bigg ] \Bigg ) v_{n+1} \Bigg ) \, e_1 \\  &= \Bigg ( \sum _{ \begin{array}{c} \varpi \in \Pi _n, \\ \forall \, I \in \varpi :\#_I\le 2 \end{array} } \left[ {\textstyle \prod ^{ \#_\varpi - 1 }_{ j=0 }} (1-2j) \right] \Bigg ( \frac{d}{d v_0} \Bigg [ \frac{ \prod ^{\#_\varpi }_{i=1} \langle Pv_{\max (I^\varpi _i) {\mathbb {1}}_{\{2\}}(\#_{I^\varpi _i})} , v_{ \min (I^\varpi _i) } \rangle _H }{ [ 1 + \Vert Pv_0 \Vert ^2_H ]^{ ( \#_\varpi - \nicefrac {1}{2} ) } } \Bigg ] \Bigg ) v_{n+1} \Bigg ) \, e_1 \\  &= \Bigg ( \sum _{ \begin{array}{c} \varpi \in \Pi _n, \\ \forall \, I \in \varpi :\#_I\le 2 \end{array} } \Bigg \{ \frac{ \big [ \prod ^{ \#_\varpi }_{ j=0 } (1-2j) \big ] \langle P v_0, P v_{n+1} \rangle _H \big [ \prod ^{ \#_\varpi }_{ i=1 } \langle Pv_{\max (I^\varpi _i) {\mathbb {1}}_{\{2\}}(\#_{I^\varpi _i})} , v_{ \min (I^\varpi _i) } \rangle _H \big ] }{ [ 1 + \Vert Pv_0 \Vert ^2_H ]^{ ( \#_\varpi + \nicefrac {1}{2} ) } } \\  &\quad + \frac{ \big [\prod ^{\#_\varpi -1}_{j=0}(1-2j)\big ] }{ [1+\Vert P v_0\Vert ^2_H]^{(\#_\varpi -\nicefrac {1}{2})} } \Bigg ( \frac{d}{dv_0} \Bigg [ \prod ^{\#_\varpi }_{i=1} \langle Pv_{\max (I^\varpi _i) {\mathbb {1}}_{\{2\}}(\#_{I^\varpi _i})} , v_{ \min (I^\varpi _i) } \rangle _H \Bigg ] \Bigg ) v_{n+1} \Bigg \} \Bigg ) e_1 . \end{aligned} \end{aligned}$$

Hence, we obtain that for all $v_0, v_1, \ldots , v_{n+1} \in H$ it holds that

28 $$\begin{aligned} \begin{aligned}&B^{(n+1)}( v_0 )( v_1,v_2,\ldots ,v_{n+1} ) \\  &= \Bigg ( \sum _{ \begin{array}{c} \varpi \in \Pi _n, \\ \forall \, I \in \varpi :\#_I\le 2 \end{array} } \Bigg \{ \frac{ \big [ \prod ^{ \#_\varpi }_{ j=0 } (1-2j) \big ] \langle P v_0, P v_{n+1} \rangle _H \big [ \prod _{ I \in \varpi } \langle Pv_{\max (I) {\mathbb {1}}_{\{2\}}(\#_I)} , v_{ \min (I) } \rangle _H \big ] }{ [ 1 + \Vert Pv_0 \Vert ^2_H ]^{ ( \#_\varpi + \nicefrac {1}{2} ) } } \\  &\quad + \sum _{i\in \{1,2,\ldots ,\#_\varpi \}} \frac{ \big [ \prod ^{ \#_\varpi - 1 }_{ j=0 } (1-2j) \big ] }{ [ 1 + \Vert Pv_0 \Vert ^2_H ]^{ ( \#_\varpi - \nicefrac {1}{2} ) } }\, \langle {\mathbb {1}}_{\{2\}}(\#_{I^\varpi _i\cup \{n+1\}}) \, Pv_{n+1} , v_{ \min (I^\varpi _i) } \rangle _H \\  &\quad \cdot \prod _{ j \in \{1,2,\ldots ,\#_\varpi \}\setminus \{i\} } \langle Pv_{\max (I^\varpi _j) {\mathbb {1}}_{\{2\}}(\#_{I^\varpi _j})} , v_{ \min (I^\varpi _j) } \rangle _H \Bigg \} \Bigg ) \, e_1 \\  &= \Bigg ( \sum _{ \begin{array}{c} \varpi \in \Pi _n \end{array} } \Bigg \{ \frac{ \big [ \prod ^{ \#_{ \varpi \cup \{ \{n+1\} \} }-1 }_{ j=0 } (1-2j) \big ] \big [ \prod _{ I \in \varpi \cup \{ \{n+1\} \} } \langle {\mathbb {1}}_{\{1,2\}}(\#_I) Pv_{\max (I) {\mathbb {1}}_{\{2\}}(\#_I)} , v_{ \min (I) } \rangle _H \big ] }{ [ 1 + \Vert Pv_0 \Vert ^2_H ]^{ ( \#_{ \varpi \cup \{ \{n+1\} \} } - \nicefrac {1}{2} ) } } \\  &\quad + \sum _{i\in \{1,2,\ldots ,\#_\varpi \}} \frac{ \big [ \prod ^{ \#_\varpi - 1 }_{ j=0 } (1-2j) \big ] }{ [ 1 + \Vert Pv_0 \Vert ^2_H ]^{ ( \#_\varpi - \nicefrac {1}{2} ) } }\, \langle {\mathbb {1}}_{\{1,2\}}(\#_{I^\varpi _i\cup \{n+1\}}) \, Pv_{n+1} , v_{ \min (I^\varpi _i) } \rangle _H \\  &\quad \cdot \prod _{ I \in \varpi \setminus \{I^\varpi _i\} } \langle {\mathbb {1}}_{\{1,2\}}(\#_I) Pv_{\max (I) {\mathbb {1}}_{\{2\}}(\#_{I})} , v_{ \min (I) } \rangle _H \Bigg \} \Bigg ) \, e_1 . \end{aligned} \end{aligned}$$

This implies that for all $v_0, v_1, \ldots , v_{n+1} \in H$ it holds that

29 $$\begin{aligned} \begin{aligned}&B^{(n+1)}( v_0 )( v_1,v_2,\ldots ,v_{n+1} )\\&\quad = \Bigg ( \sum _{ \varpi \in \Pi _n } \Bigg \{ \sum _{ \begin{array}{c} \Xi \in \Pi _{n+1}, \\ \Xi = \varpi \cup \{\{n+1\}\} \end{array} } \Bigg [ \frac{ \big [ \prod ^{ \#_\Xi - 1 }_{ i=0 } (1-2i) \big ] \big [ \prod _{ I \in \Xi } \langle \mathbb {1}_{\{1,2\}}( \#_I ) \, Pv_{\max (I) {\mathbb {1}}_{\{2\}}(\#_I)} , v_{ \min (I) } \rangle _H \big ] }{ [ 1 + \Vert Pv_0 \Vert ^2_H ]^{ ( \#_\Xi - \nicefrac {1}{2} ) } } \Bigg ]\\&\qquad + \sum _{\begin{array}{c} \Xi \in \Pi _{n+1}, \, i \in \{1,2,\ldots ,\#_\varpi \}, \\ \Xi = (\varpi \setminus \{I^\varpi _i\})\cup \{I^\varpi _i\cup \{n+1\}\} \end{array}} \Bigg [ \frac{ \big [ \prod ^{ \#_\Xi - 1 }_{ i=0 } (1-2i) \big ] \big [ \prod _{ I \in \Xi } \langle \mathbb {1}_{\{1,2\}}( \#_I ) \, Pv_{\max (I) {\mathbb {1}}_{\{2\}}(\#_I)} , v_{ \min (I) } \rangle _H \big ] }{ [ 1 + \Vert Pv_0 \Vert ^2_H ]^{ ( \#_\Xi - \nicefrac {1}{2} ) } } \Bigg ] \Bigg \} \Bigg ) \, e_1. \end{aligned} \end{aligned}$$

Combining this with the fact that

30 $$\begin{aligned} \begin{aligned}&\Pi _{n+1} = \Big \{ \varpi \cup \big \{\{n+1\}\big \} :\varpi \in \Pi _n \Big \} \\  &\biguplus \Big \{ \big \{ I^\varpi _1, I^\varpi _2, \ldots , I^\varpi _{i-1}, I^\varpi _i \cup \{n+1\}, I^\varpi _{i+1}, I^\varpi _{i+2}, \ldots , I^\varpi _{\#_\varpi } \big \} :i \in \{1,2,\ldots ,\#_\varpi \}, \\  &\varpi \in \Pi _n \Big \} \end{aligned} \end{aligned}$$

proves (23) in the case $n+1$. Induction therefore establishes item (ii).

It thus remains to prove item (iii). For this we note that for all $n \in {\mathbb {N}}$, $\varpi \in \Pi _n$ with $\forall \, I \in \varpi :\#_I \le 2$ it holds that

31 $$\begin{aligned} \#_{\{ I \in \varpi :\#_I = 1 \}} = 2\#_\varpi -n . \end{aligned}$$

Next observe that the Cauchy-Schwarz inequality and (23) ensure that for all $n \in {\mathbb {N}}$, $v_0, v_1, \ldots , v_n \in H$ it holds that

32 $$\begin{aligned} \begin{aligned}&\Vert B^{(n)}( v_0 )( v_1,v_2,\ldots ,v_n ) \Vert _{ H }\\&\quad = \Bigg \Vert \Bigg ( {\sum \limits _{ \varpi \in \Pi _n }} \frac{ \big [ {\prod ^{ \#_\varpi - 1 }_{ i=0 }} (1-2i) \big ] \big [ \prod \nolimits _{ I \in \varpi } \langle \mathbb {1}_{\{1,2\}}( \#_I ) \, Pv_{\max (I) {\mathbb {1}}_{\{2\}}(\#_I)} , v_{ \min (I) } \rangle _H \big ] }{ \big [ 1 + \Vert Pv_0 \Vert ^2_H \big ]^{ ( \#_\varpi - \nicefrac {1}{2} ) } } \Bigg ) \, e_1 \Bigg \Vert _H \\&\quad = \Bigg | {\sum _{ \varpi \in \Pi _n, \, \forall \, I \in \varpi :\#_{I} \le 2 }} \frac{ \big [ {\prod ^{ \#_\varpi - 1 }_{ i=0 }} (1-2i) \big ] \big [ \prod \nolimits _{ I \in \varpi } \langle Pv_{\max (I) {\mathbb {1}}_{\{2\}}(\#_I)} , Pv_{ \min (I) } \rangle _H \big ] }{ \big [ 1 + \Vert Pv_0 \Vert ^2_H \big ]^{ ( \#_\varpi - \nicefrac {1}{2} ) } } \Bigg |\\&\quad \le {\sum _{ \varpi \in \Pi _n, \, \forall \, I \in \varpi :\#_{I} \le 2 }} \frac{ \big | {\prod ^{ \#_\varpi - 1 }_{ i=0 }} (1-2i) \big | \prod \nolimits _{ I \in \varpi } \big [ \Vert Pv_{\max (I) {\mathbb {1}}_{\{2\}}(\#_I)} \Vert _H \, \Vert Pv_{ \min (I) } \Vert _H \big ] }{ \big [ 1 + \Vert Pv_0 \Vert ^2_H \big ]^{ ( \#_\varpi - \nicefrac {1}{2} ) } } . \end{aligned} \end{aligned}$$

Moreover, the fact that $\forall \, n \in {\mathbb {N}}, \, \varpi \in \Pi _n :\cup _{I\in \varpi } I = \{1,2,\ldots ,n\}$ implies that for all $n \in {\mathbb {N}}$, $\varpi \in \Pi _n$, $v_0, v_1, \ldots , v_n \in H$ with $\forall \, I \in \varpi :\#_{I} \le 2$ it holds that

33 $$\begin{aligned} \begin{aligned}&\prod \nolimits _{ I \in \varpi } \big [ \Vert Pv_{\max (I) {\mathbb {1}}_{\{2\}}(\#_I)} \Vert _H \, \Vert Pv_{ \min (I) } \Vert _H \big ]\\&\quad = \bigg ( \prod \nolimits _{\begin{array}{c} I \in \varpi ,\\ \#_I = 1 \end{array}} \big [ \Vert Pv_0 \Vert _H \, \Vert Pv_{ \min (I) } \Vert _H \big ] \bigg ) \, \bigg ( \prod \nolimits _{\begin{array}{c} I \in \varpi ,\\ \#_I = 2 \end{array}} \big [ \Vert Pv_{\max (I)} \Vert _H \, \Vert Pv_{ \min (I) } \Vert _H \big ] \bigg )\\&\quad = \bigg ( \prod \nolimits _{\begin{array}{c} I \in \varpi ,\\ \#_I = 1 \end{array}} \Vert Pv_0 \Vert _H \bigg ) \, \bigg \{ \bigg ( \prod \nolimits _{\begin{array}{c} I \in \varpi ,\\ \#_I = 1 \end{array}} \Vert Pv_{\min (I)} \Vert _H \bigg ) \, \bigg ( \prod \nolimits _{\begin{array}{c} I \in \varpi ,\\ \#_I = 2 \end{array}} \big [ \Vert Pv_{\max (I)} \Vert _H \, \Vert Pv_{ \min (I) } \Vert _H \big ] \bigg ) \bigg \}\\&\quad = \Vert Pv_0\Vert ^{ \#_{\{I\in \varpi :\#_I=1\}} }_H \, \prod ^{ n }_{ i=1 } \Vert Pv_i \Vert _H . \end{aligned} \end{aligned}$$

This, (31), and (32) show that for all $n \in {\mathbb {N}}$, $v_0, v_1, \ldots , v_n \in H$ it holds that

34 $$\begin{aligned} \begin{aligned}&\Vert B^{(n)}( v_0 )( v_1,v_2,\ldots ,v_n ) \Vert _{ H } \\&\quad \le \sum _{ \varpi \in \Pi _n, \, \forall \, I \in \varpi :\#_{I} \le 2 } \left| \prod ^{ \#_\varpi - 1 }_{ i=0 } (1-2i) \right| \frac{ \Vert Pv_0\Vert ^{ \#_{\{I\in \varpi :\#_I=1\}} }_H }{ [ 1 + \Vert Pv_0 \Vert ^2_H ]^{ ( \#_\varpi - 1/2 ) } } \left[ \prod ^{ n }_{ i=1 } \Vert Pv_i \Vert _H \right] \\  &\quad = \sum _{ \varpi \in \Pi _n, \, \forall \, I \in \varpi :\#_{I} \le 2 } \left| \prod ^{ \#_\varpi - 1 }_{ i=0 } (1-2i) \right| \frac{ \Vert Pv_0\Vert ^{ (2\#_\varpi - n) }_H }{ [ 1 + \Vert Pv_0 \Vert ^2_H ]^{ ( \#_\varpi - 1/2 ) } } \left[ \prod ^{ n }_{ i=1 } \Vert Pv_i \Vert _H \right] . \end{aligned} \end{aligned}$$

The fact that $\forall \, v \in H :\Vert Pv\Vert _H \le \Vert v\Vert _H$ therefore implies that for all $n \in {\mathbb {N}}$ it holds that

35 $$\begin{aligned} \begin{aligned}&\sup _{ v \in H } \Vert B^{(n)}( v ) \Vert _{ L^{(n)}( H, H ) } \\  &\quad \le \sum _{ \varpi \in \Pi _n, \, \forall \, I \in \varpi :\#_{I} \le 2 } \left| \prod ^{ \#_\varpi - 1 }_{ i=0 } (1-2i) \right| \sup _{ v \in H } \bigg [ \frac{ \Vert Pv\Vert ^{ (2\#_\varpi - n) }_H }{ [ 1 + \Vert Pv \Vert ^2_H ]^{ ( \#_\varpi - 1/2 ) } } \bigg ] \\  &\quad \le \sum _{ \varpi \in \Pi _n, \, \forall \, I \in \varpi :\#_{I} \le 2 } \prod ^{ \#_\varpi - 1 }_{ i=0 } |2i-1| \le \sum _{\varpi \in \Pi _n} [2\#_\varpi ]^{\#_\varpi } . \end{aligned} \end{aligned}$$

This and the fact that $\forall \, n \in {\mathbb {N}}, \, \varpi \in \Pi _n :\#_{\Pi _n}+\#_\varpi < \infty$ establish item (iii). The proof of Lemma 2.1 is thus completed.

### Explicit representations for the derivative processes

In this section we present in item (v) of Lemma 2.2 below suitable explicit representation formulas for the derivatives of solutions of a certain class of SEEs with respect to their initial values. More specifically, in the setting of Sect. 2.1 we provide in item (v) of Lemma 2.2 below for every $n\in {\mathbb {N}}$ an explicit representation formula for the *n*-th derivative of the solution process of the SEE

36 $$\begin{aligned} dX_t = AX_t \, dt + B(X_t) \, dW_t \end{aligned}$$

with respect to its initial value where $A:D(A)\subseteq H \rightarrow H$, $B:H\rightarrow H$, and $W:[0,T]\times \Omega \rightarrow {\mathbb {R}}$ are specified in Sect. 2.1 above. In the following we briefly sketch some of the key facts we employ in the proof of item (v) of Lemma 2.2 below. In the setting of Sect. 2.1 above observe that the fact that for all $v\in H$ it holds that

37 $$\begin{aligned} P v = \sum _{ n = 2 }^{ \infty } \langle e_n, v \rangle _H e_n \end{aligned}$$

(see (9) above) and the fact that for all $n \in {\mathbb {N}}$, $v_0, v_1, \ldots , v_n \in H$ there exists a real number $C\in {\mathbb {R}}$ such that

38 $$\begin{aligned} B^{(n)}(v_0)(v_1,v_2,\ldots ,v_n) = C\cdot e_1 \end{aligned}$$

(cf. (23) above) imply that for all $n\in {\mathbb {N}}$, $v_0,v_1,\ldots ,v_n\in H$ it holds that

39 $$\begin{aligned} PB^{(n)}(v_0)(v_1,v_2,\ldots ,v_n) = 0. \end{aligned}$$

Combining this with the general implicit representation formula for derivatives of the solutions of SEE (36) in Andersson et al. [3, item (i) of Theorem 2.1] allows us to establish item (v) of Lemma 2.2 below.

#### Lemma 2.2

(Exact formulas of derivative processes) Assume the setting in Sect. 2.1. Then

(i) there exist up-to-modifications unique $$ ( \mathcal {F}_t )_{ t \in [0,T] } $$/-predictable stochastic processes $$ X^{0,x} :$$ $$ [0,T] \times \Omega \rightarrow H $$, $$ x \in H $$, such that for all $$ p \in [2,\infty ) $$, $$ x \in H $$, $$ t \in [0,T] $$ it holds that $$ \sup _{ s \in [0,T] } {\mathbb {E}}\big [ \Vert X_s^{0,x} \Vert ^p_H \big ] < \infty $$ and
  40 $$\begin{aligned} \begin{aligned}&|\![X_t^{0,x} - e^{tA} x]\!| = \int _0^t e^{ ( t - s ) A } B(X_s^{0,x}) \, dW_s , \end{aligned} \end{aligned}$$
(ii) it holds for all $p \in [2,\infty )$, $t \in [0,T]$ that $H \ni x \mapsto |\![X^{0,x}_t]\!| \in L^{p}({\mathbb {P}};H)$ is infinitely often Fréchet differentiable,
(iii) there exist up-to-modifications unique /-predictable stochastic processes $X^{n,\textbf{u}} :$ $[0,T] \times \Omega \rightarrow H$, $\textbf{u} \in H^{n+1}$, $n \in {\mathbb {N}}$, such that for all $p \in [2,\infty )$, $n \in {\mathbb {N}}$, , $t \in [0,T]$ it holds that 41
(iv) it holds for all $p \in [2,\infty )$, $n \in {\mathbb {N}}$, $\delta _1,\delta _2,\ldots ,\delta _n \in [0,\infty )$, $t \in (0,T]$ with $\sum ^n_{i=1} \delta _i < \nicefrac {1}{2}$ that
  42 $$\begin{aligned} \sup _{ \textbf{u} = (u_0,u_1,\ldots ,u_n) \in H \times ({H}\setminus \{0\})^n } \frac{ \big ({\mathbb {E}}\big [ \Vert X^{ n,\textbf{u} }_t \Vert ^p_H \big ]\big )^{\nicefrac {1}{p}} }{ \prod ^{ n }_{ i=1 } \Vert u_i\Vert _{H_{-\delta _i}} } < \infty , \end{aligned}$$
  and
(v) it holds for all $n \in {\mathbb {N}}_0$, $\textbf{u} = ( u_0, u_1, \ldots , u_n ) \in H^{ n+1 }$, $t \in [0,T]$ that
  43 $$\begin{aligned} \begin{aligned}&|\![X_t^{n,\textbf{u}} \!-\! \mathbb {1}_{ \{ 0, 1 \} }(n) \, e^{tA} u_n ]\!| \!=\! \int _0^t e^{ ( t \!-\! s ) A } B^{ ( n ) }( e^{sA} u_0 ) \big ( e^{sA} u_1 , e^{sA} u_2 , \dots , e^{sA} u_n \big ) \, dW_s . \end{aligned} \end{aligned}$$

#### Proof

Throughout this proof for every $n \in {\mathbb {N}}$, $\varpi \in \Pi _n$ let $I^\varpi _i \in \varpi$, $i \in \{1,2,\ldots ,\#_\varpi \}$, satisfy that

44 $$\begin{aligned} \min ( I^\varpi _1 )< \min ( I_2^\varpi )< \dots < \min ( I_{ \#_\varpi }^{ \varpi } ), \end{aligned}$$

let $I_{ i, j }^\varpi \in I_i^{ \varpi } \subseteq {\mathbb {N}}$, $j \in \{ 1,2,\ldots ,\#_{I^\varpi _i} \}$, $i \in \{1,2,\ldots ,\#_\varpi \}$, satisfy for all $i \in \{1,2,\ldots ,\#_\varpi \}$ that

45 $$\begin{aligned} I_{ i, 1 }^\varpi< I_{ i, 2 }^\varpi< \dots < I_{ i, \#_{ I_i^{ \varpi } } }^\varpi , \end{aligned}$$

and let $[ \cdot ]_i^\varpi :H^{ n + 1 } \rightarrow H^{ \#_{I_i^\varpi } + 1 }$, $i \in \{ 1, 2, \dots , \#_\varpi \}$, satisfy for all $i \in \{ 1, 2, \dots , \#_\varpi \}$, $\textbf{u} = (u_0, u_1, \dots , u_n) \in H^{ n + 1 }$ that

46 $$\begin{aligned} [ \textbf{u} ]_i^\varpi = ( u_0, u_{ I_{ i, 1 }^\varpi } , u_{ I_{ i, 2 }^\varpi } , \dots , u_{ I_{ i, \#_{I_i^\varpi } }^\varpi } ). \end{aligned}$$

We note that items (i) and (iii) of Lemma 2.1 and items (i), (ii), (ix), and (x) of Theorem 2.1 in Andersson et al. [3] (with $T=T$, $\eta =0$, $H=H$, $U={\mathbb {R}}$, $W=W$, $A=A$, $n=n$, $F=(H\ni v \mapsto 0 \in H)$, $B=(H\ni v \mapsto ({\mathbb {R}}\ni u \mapsto uB(v) \in H) \in HS({\mathbb {R}},H))$, $\alpha =0$, $\beta =0$ for $n \in {\mathbb {N}}$ in the notation of Theorem 2.1 in [3]) ensure that

- there exist up-to-modifications unique /-predictable stochastic processes $X^{ n,\textbf{u} } :$ $[ 0, T ] \times \Omega \rightarrow H$, $\textbf{u} \in H^{n+1}$, $n \in {\mathbb {N}}_0$, such that for all $n \in {\mathbb {N}}_0$, $p \in [2,\infty )$, $\textbf{u} = (u_0,u_1,\ldots ,u_n) \in H^{n+1}$, $t \in [0,T]$ it holds that $\sup _{s\in [0,T]} {\mathbb {E}}\big [\Vert X^{n,\textbf{u}}_s\Vert ^p_H\big ] < \infty$ and
  47 $$\begin{aligned}  &   |\![X_t^{n,\textbf{u}} - \mathbb {1}_{ \{ 0, 1 \} }(n) \, e^{tA} u_n ]\!| = \int _0^t e^{ ( t - s ) A } \Bigg [ \mathbb {1}_{ \{ 0 \} }(n) \, B(X_s^{0,u_0}) \nonumber \\  &   \quad + \sum _{ \varpi \in \Pi _n } B^{ ( \#_\varpi ) }( X_s^{ 0,u_0 } ) \big ( X_s^{ \#_{I^\varpi _1}, [ \textbf{u} ]_1^{ \varpi } }, X_s^{ \#_{I^\varpi _2}, [ \textbf{u} ]_2^{ \varpi } }, \dots , X_s^{ \#_{I^\varpi _{\#_\varpi }}, [\textbf{u} ]_{ \#_\varpi }^{ \varpi } } \big ) \Bigg ] \, dW_s,\nonumber \\ \end{aligned}$$
- it holds for all $p \in [2,\infty )$, $t \in [0,T]$ that $H \ni x \mapsto |\![X^{0,x}_t]\!| \in L^{p}({\mathbb {P}};H)$ is infinitely often Fréchet differentiable,
- it holds for all $n \in {\mathbb {N}}$, $p \in [2,\infty )$, $\textbf{u} \in H^n$, $x \in H$, $t \in [0,T]$ that
  48 $$\begin{aligned} \begin{aligned}&\big ( \tfrac{d^n}{dx^n} |\![X^{0,x}_t]\!| \big ) \textbf{u} = \big ( H \ni y \mapsto |\![X^{0,y}_t]\!| \in L^{p}({\mathbb {P}};H) \big )^{(n)}(x) \, \textbf{u} = |\![X^{n,(x,\textbf{u})}_t]\!| , \end{aligned} \end{aligned}$$
  and
- it holds for all $n \in {\mathbb {N}}$, $p \in [2,\infty )$, $\delta _1,\delta _2,\ldots ,\delta _n \in [0,\infty )$, $t \in (0,T]$ with $\sum ^n_{i=1} \delta _i < \nicefrac {1}{2}$ that
  49 $$\begin{aligned} \sup _{ \textbf{u} = (u_0,u_1,\ldots ,u_n) \in H \times ({H}\setminus \{0\})^n } \frac{ \big ({\mathbb {E}}\big [ \Vert X^{ n,\textbf{u} }_t \Vert ^p_H \big ]\big )^{\nicefrac {1}{p}} }{ \prod ^{ n }_{ i=1 } \Vert u_i\Vert _{H_{-\delta _i}} } < \infty . \end{aligned}$$

This, items (i) and (iii) of Lemma 2.1, and item (i) of Corollary 2.10 in Andersson et al. [2] establish items (i)–(iv). It thus remains to prove (v). Note that (47) and the fact that $\forall \, v \in H :P(B(v)) = 0$ imply that for all $x \in H$, $t \in [0,T]$ it holds that

50 $$\begin{aligned} |\![ P X^{0,x}_t - P e^{tA} x]\!|= &   P \int ^t_0 e^{(t-s)A} B( X^{0,x}_s ) \, dW_s\nonumber \\= &   \int ^t_0 e^{(t-s)A} \big [ P \big ( B( X^{0,x}_s ) \big ) \big ] \, dW_s = 0 . \end{aligned}$$

This shows that for all $x \in H$, $t \in [0,T]$ it holds that

51 $$\begin{aligned} {\mathbb {P}}\bigg ( B( X^{0,x}_t ) = \sqrt{ 1 + \Vert P X^{0,x}_t \Vert ^2_H } \, e_1 = \sqrt{ 1 + \Vert P e^{tA} x \Vert ^2_H } \, e_1 = B( e^{tA} x ) \bigg ) =1 . \end{aligned}$$

This and (47) yield that for all $x \in H$, $t \in [0,T]$ it holds that

52 $$\begin{aligned} |\![X^{0,x}_t-e^{tA} x]\!| = \int ^t_0 e^{ (t-s)A } B( e^{sA} x ) \, dW_s . \end{aligned}$$

Next note that item (ii) of Lemma 2.1 ensures that for all $n \in {\mathbb {N}}$, $\textbf{v} \in H^n$, $x \in H$ it holds that $P \big (B^{(n)}(x)\textbf{v} \big ) = 0$. This and (47) imply that for all $n \in {\mathbb {N}}$, $\textbf{u} = (u_0,u_1,\ldots ,u_n) \in H^{n+1}$, $t \in [0,T]$ it holds that

53 $$\begin{aligned} \begin{aligned}&|\![P X^{n,\textbf{u}}_t - {\mathbb {1}}_{\{1\}}(n) \, P e^{tA} u_1]\!| \\&\quad = P \int ^t_0 e^{(t-s)A} \sum _{ \varpi \in \Pi _n } B^{ ( \#_\varpi ) }( X_s^{ 0,u_0 } ) \big ( X_s^{ \#_{I^\varpi _1}, [ \textbf{u} ]_1^{ \varpi } } , X_s^{ \#_{I^\varpi _2}, [ \textbf{u} ]_2^{ \varpi } } , \dots , X_s^{ \#_{I^\varpi _{\#_\varpi }}, [\textbf{u} ]_{ \#_\varpi }^{ \varpi } } \big ) \, dW_s \\&\quad = \int ^t_0 e^{(t-s)A} \sum _{ \varpi \in \Pi _n } \bigg [ P\bigg ( B^{ ( \#_\varpi ) }( X_s^{ 0,u_0 } ) \big ( X_s^{ \#_{I^\varpi _1}, [ \textbf{u} ]_1^{ \varpi } } , X_s^{ \#_{I^\varpi _2}, [ \textbf{u} ]_2^{ \varpi } } , \dots , X_s^{ \#_{I^\varpi _{\#_\varpi }}, [\textbf{u} ]_{ \#_\varpi }^{ \varpi } } \big ) \bigg ) \bigg ] \, dW_s =0 . \end{aligned} \end{aligned}$$

Hence, we obtain that for all $n\in \{2,3,\ldots \}$, $\textbf{u}\in H^{n+1}$, $t\in [0,T]$ it holds that

54 $$\begin{aligned} {\mathbb {P}}\big ( P(X^{n,\textbf{u}}_t) = 0 \big ) = 1 . \end{aligned}$$

In addition, note that item (ii) of Lemma 2.1 implies that for all $n\in {\mathbb {N}}$, $v_0,v_1,\ldots ,v_n \in H$ it holds that

55 $$\begin{aligned} B^{(n)}(v_0)(v_1,v_2,\ldots ,v_n)= B^{(n)}(Pv_0)(Pv_1,Pv_2,\ldots ,Pv_n) . \end{aligned}$$

Combining this with (54) ensures that for all $n \in {\mathbb {N}}$, $\varpi \in \Pi _n$, $\textbf{u} = (u_0,u_1,\ldots ,u_n) \in H^{n+1}$, $t \in [0,T]$ with $\varpi \ne \big \{ \{1\}, \{2\}, \ldots , \{n\} \big \}$ it holds that

56 $$\begin{aligned} \begin{aligned}&{\mathbb {P}}\bigg ( B^{ ( \#_\varpi ) }( X_t^{ 0, u_0 } ) \big ( X_t^{ \#_{I^\varpi _1}, [ \textbf{u} ]_1^{ \varpi } } , X_t^{ \#_{I^\varpi _2}, [ \textbf{u} ]_2^{ \varpi } } , \dots , X_t^{ \#_{I^\varpi _{\#_\varpi }}, [\textbf{u} ]_{ \#_\varpi }^{ \varpi } } \big ) \\  &\quad = B^{ ( \#_\varpi ) }( P X_t^{ 0, u_0 } ) \big ( P X_t^{ \#_{I^\varpi _1}, [ \textbf{u} ]_1^{ \varpi } } , P X_t^{ \#_{I^\varpi _2}, [ \textbf{u} ]_2^{ \varpi } } , \dots , P X_t^{ \#_{I^\varpi _{\#_\varpi }}, [\textbf{u} ]_{ \#_\varpi }^{ \varpi } } \big ) = 0 \bigg ) =1. \end{aligned} \end{aligned}$$

Equation (55) hence implies that for all $n \in {\mathbb {N}}$, $\textbf{u} = (u_0,u_1,\ldots ,u_n) \in H^{n+1}$, $t \in [0,T]$ it holds that

57 $$\begin{aligned} \begin{aligned}&{\mathbb {P}}\Bigg ( \sum _{ \varpi \in \Pi _n } B^{ ( \#_\varpi ) }( X_t^{ 0, u_0 } ) \big ( X_t^{ \#_{I^\varpi _1}, [ \textbf{u} ]_1^{ \varpi } } , X_t^{ \#_{I^\varpi _2}, [ \textbf{u} ]_2^{ \varpi } } , \dots , X_t^{ \#_{I^\varpi _{\#_\varpi }}, [\textbf{u} ]_{ \#_\varpi }^{ \varpi } } \big ) \\  &\quad = B^{ (n) }( X_t^{ 0, u_0 } ) \big ( X_t^{ 1, (u_0,u_1) } , X_t^{ 1, (u_0,u_2) } , \dots , X_t^{ 1, (u_0,u_n) } \big ) \\  &\quad = B^{ (n) }( P X_t^{ 0, u_0 } ) \big ( P X_t^{ 1, (u_0,u_1) } , P X_t^{ 1, (u_0,u_2) } , \dots , P X_t^{ 1, (u_0,u_n) } \big ) \Bigg )=1 . \end{aligned} \end{aligned}$$

Combining this with (50), (53), and (55) shows that for all $n \in {\mathbb {N}}$, $\textbf{u} = (u_0,u_1,\ldots ,u_n) \in H^{n+1}$, $t \in [0,T]$ it holds that

58 $$\begin{aligned}&{\mathbb {P}}\Bigg ( \sum _{ \varpi \in \Pi _n } B^{ ( \#_\varpi ) }( X_t^{ 0, u_0 } ) \big ( X_t^{ \#_{I^\varpi _1}, [ \textbf{u} ]_1^{ \varpi } } , X_t^{ \#_{I^\varpi _2}, [ \textbf{u} ]_2^{ \varpi } } , \dots , X_t^{ \#_{I^\varpi _{\#_\varpi }}, [\textbf{u} ]_{ \#_\varpi }^{ \varpi } } \big )\nonumber \\  &\quad = B^{ ( n ) }( P e^{tA} { u_0 } ) \big ( P e^{tA} u_1 , P e^{tA} u_2 , \dots , P e^{tA} u_n \big )\nonumber \\  &\quad = B^{ ( n ) }( e^{tA} { u_0 } ) \big ( e^{tA} u_1 , e^{tA} u_2 , \dots , e^{tA} u_n \big ) \Bigg )=1 . \end{aligned}$$

This and (47) assure that for all $n \in {\mathbb {N}}$, $\textbf{u} = (u_0,u_1,\ldots ,u_n) \in H^{n+1}$, $t \in [0,T]$ it holds that

59 $$\begin{aligned} \begin{aligned}&|\![X_t^{n,\textbf{u}} - \mathbb {1}_{ \{ 1 \} }(n) \, e^{tA} u_n ]\!| = \int _0^t e^{ ( t - s ) A } B^{ ( n ) }( e^{sA} u_0 ) \big ( e^{sA} u_1 , e^{sA} u_2 , \dots , e^{sA} u_n \big ) \, dW_s . \end{aligned} \end{aligned}$$

Combining this and (52) establishes item (v). The proof of Lemma 2.2 is thus completed.

Let us add a few comments regarding a part of the argumentation in the proof of item (v) of Lemma 2.2 above. Roughly speaking, in (53)–(59) above we employ the derivative formula in Andersson et al. [3, item (i) of Theorem 2.1] (see (47) in the proof of Lemma 2.2 above) and the fact that $Pe_1=0$ to establish item (v) of Lemma 2.2 above. We note that an alternative procedure of proving item (v) of Lemma 2.2 above is for every $n\in {\mathbb {N}}$ to differentiate *n*-times both sides of (52) above.

### Disproof of regularities for the derivative processes

#### Theorem 2.1

Assume the setting in Sect. 2.1, let $c \in (0,\infty )$, and assume for all $n \in {\mathbb {N}}$ that $\lambda _n = -c n^2$. Then

(i) there exist up-to-modifications unique /-predictable stochastic processes $$ X^{0,x} :$$ $$ [0,T] \times \Omega \rightarrow H $$, $$ x \in H $$, such that for all $$ p \in [2,\infty ) $$, $$ x \in H $$, $$ t \in [0,T] $$ it holds that $$ \sup _{ s \in [0,T] } {\mathbb {E}}\big [\Vert X_s^{0,x} \Vert ^p_H \big ] < \infty $$ and
  60 $$\begin{aligned} \begin{aligned}&|\![X_t^{0,x} - e^{tA} x]\!| = \int _0^t e^{ ( t - s ) A } B(X_s^{0,x}) \, dW_s , \end{aligned} \end{aligned}$$
(ii) it holds for all $p \in [2,\infty )$, $t \in [0,T]$ that $H \ni x \mapsto |\![X^{0,x}_t]\!| \in L^{p}({\mathbb {P}};H)$ is infinitely often Fréchet differentiable,
(iii) there exist up-to-modifications unique /-predictable stochastic processes $X^{n,\textbf{u}} :$ $[0,T] \times \Omega \rightarrow H$, $\textbf{u} \in H^{n+1}$, $n \in {\mathbb {N}}$, such that for all $p \in [2,\infty )$, $n \in {\mathbb {N}}$, $\textbf{u} \in H^n$, $x \in H$, $t \in [0,T]$ it holds that
  61 $$\begin{aligned} \begin{aligned}&\big ( \tfrac{d^n}{dx^n} |\![X^{0,x}_t]\!| \big ) \textbf{u} = \big ( H \ni y \mapsto |\![X^{0,y}_t]\!| \in L^{p}({\mathbb {P}};H) \big )^{(n)}(x) \, \textbf{u} = |\![X^{n,(x,\textbf{u})}_t]\!| , \end{aligned} \end{aligned}$$
(iv) it holds for all $p \in [2,\infty )$, $n \in {\mathbb {N}}$, $q,\delta _1,\delta _2,\ldots ,\delta _n \in [0,\infty )$, $t \in (0,T]$ with $\sum ^n_{i=1} \delta _i < \nicefrac {1}{2}$ that
  62 $$\begin{aligned} \sup _{ \textbf{u} = (u_0,u_1,\ldots ,u_n) \in H \times ({H}\setminus \{0\})^n } \frac{ \big ({\mathbb {E}}\big [ \Vert X^{ n,\textbf{u} }_t \Vert ^p_{H_{-q}} \big ]\big )^{\nicefrac {1}{p}} }{ \prod ^{ n }_{ i=1 } \Vert u_i\Vert _{H_{-\delta _i}} } < \infty , \end{aligned}$$
  and
(v) it holds for all $p \in [2,\infty )$, $n \in {\mathbb {N}}$, $q\in [0,\infty )$, $\delta _1, \delta _2, \ldots , \delta _n \in {\mathbb {R}}$, $t \in (0,T]$ with $\sum ^n_{i=1} \delta _i > \nicefrac {1}{2}$ that
  63 $$\begin{aligned} \sup _{ \textbf{u}=(u_0,u_1,\ldots ,u_n) \in ({(\cap _{r\in {\mathbb {R}}}H_r)}\setminus \{0\})^{n+1} } \frac{ \big ({\mathbb {E}}\big [ \Vert X^{ n,\textbf{u} }_t \Vert ^p_{H_{-q}} \big ]\big )^{\nicefrac {1}{p}} }{ \prod ^{ n }_{ i=1 } \Vert u_i\Vert _{H_{-\delta _i}} } = \infty . \end{aligned}$$

#### Proof

Throughout this proof let $v^{ k, r }_{ n,N } \in H$, $N,n \in {\mathbb {N}}$, $k \in {\mathbb {N}}_0$, $r \in {\mathbb {R}}$, satisfy for all $N,n \in {\mathbb {N}}$, $k \in {\mathbb {N}}_0$, $r \in {\mathbb {R}}$ that

64 $$\begin{aligned} v^{ k, r }_{ n,N } = (-A)^r \left[ \sum ^N_{ j=1 } e_{k+jn} \right] = \sum ^N_{ j=1 } [ c \, (k+jn)^2 ]^r \, e_{k+jn} = c^r \left[ \sum ^N_{j=1} (k+jn)^{2r} \, e_{k+jn} \right] , \end{aligned}$$

let $\textbf{u}^{\varepsilon ,m,\varvec{\delta }}_{n,N} \in H^{n+1}$, $\varvec{\delta } \in {\mathbb {R}}^n$, $\varepsilon \in {\mathbb {R}}$, $m \in {\mathbb {N}}_0$, $N,n \in {\mathbb {N}}$, satisfy for all $N,n \in {\mathbb {N}}$, $m \in {\mathbb {N}}_0$, $\varepsilon , \delta _1,\delta _2,\ldots ,\delta _{2n+1} \in {\mathbb {R}}$ that

65 $$\begin{aligned}  &   \textbf{u}^{\varepsilon ,m,\delta _1}_{1,N} = (v^{1,-\varepsilon }_{N^m,N}, N^m \, v^{1,\delta _1-(\nicefrac {1}{2})-\varepsilon }_{N^m,N}), \end{aligned}$$

66 $$\begin{aligned}  &   \textbf{u}^{\varepsilon ,m,(\delta _1,\delta _2,\ldots ,\delta _{2n})}_{2n,N} = ( e_1, v^{1,\delta _1-\varepsilon }_{nN^m,N}, v^{1,\delta _2-\varepsilon }_{nN^m,N}, v^{2,\delta _3-\varepsilon }_{nN^m,N}, v^{2,\delta _4-\varepsilon }_{nN^m,N}, \ldots , \nonumber \\  &   v^{n-1,\delta _{2n-3}-\varepsilon }_{nN^m,N}, v^{n-1,\delta _{2n-2}-\varepsilon }_{nN^m,N}, v^{n,\delta _{2n-1}-\varepsilon }_{nN^m,N}, N^m \, v^{n,\delta _{2n}-(\nicefrac {1}{2})-\varepsilon }_{nN^m,N} ), \end{aligned}$$

and

67 $$\begin{aligned}  &   \!\!\! \textbf{u}^{\varepsilon ,m,(\delta _1,\delta _2,\ldots ,\delta _{2n+1})}_{2n+1,N} = ( v^{n+1,-\varepsilon }_{(n+1)N^m,N}, v^{1,\delta _1-\varepsilon }_{(n+1)N^m,N}, v^{1,\delta _2-\varepsilon }_{(n+1)N^m,N}, v^{2,\delta _3-\varepsilon }_{(n+1)N^m,N}, v^{2,\delta _4-\varepsilon }_{(n+1)N^m,N}, \ldots , \nonumber \\  &   v^{n,\delta _{2n-1}-\varepsilon }_{(n+1)N^m,N}, v^{n,\delta _{2n}-\varepsilon }_{(n+1)N^m,N}, N^m \, v^{n+1,\delta _{2n+1}-(\nicefrac {1}{2})-\varepsilon }_{(n+1)N^m,N} ), \end{aligned}$$

let $\theta ^n_i :H^n \rightarrow H$, $i \in \{1,2,\ldots ,n\}$, $n \in {\mathbb {N}}$, satisfy for all $n \in {\mathbb {N}}$, $i \in \{1,2,\ldots ,n\}$, $\textbf{u}=(u_1,u_2,\ldots ,u_n) \in H^n$ that $\theta ^n_i(\textbf{u}) = u_i$, let $\textbf{B}_n :[0,T] \times H^{n+1} \rightarrow H$, $n\in {\mathbb {N}}$, satisfy for all $n\in {\mathbb {N}}$, $t\in [0,T]$, $\textbf{u}=(u_0,u_1,\ldots ,u_n) \in H^{n+1}$ that

68 $$\begin{aligned} \textbf{B}_n(t,\textbf{u}) = B^{(n)}(e^{tA}u_0)(e^{tA}u_1,e^{tA}u_2,\ldots ,e^{tA}u_n) , \end{aligned}$$

and let $\lfloor \cdot \rfloor :{\mathbb {R}}\rightarrow {\mathbb {R}}$ and $\lceil \cdot \rceil :{\mathbb {R}}\rightarrow {\mathbb {R}}$ satisfy for all $t \in {\mathbb {R}}$ that

69 $$\begin{aligned} \lfloor t \rfloor = \max \!\left( (-\infty ,t] \cap \{ 0, 1 , - 1 , 2 , - 2 , \dots \} \right) = \max ( (-\infty ,t] \cap \mathbb {Z} ) \end{aligned}$$

and

70 $$\begin{aligned} \begin{aligned} \lceil t \rceil = \min \!\left( [ t, \infty ) \cap \{ 0, 1 , - 1 , 2 , - 2 , \dots \} \right) = \min ( [t,\infty ) \cap \mathbb {Z} ) . \end{aligned} \end{aligned}$$

Note that for all $N,n\in {\mathbb {N}}$, $m\in {\mathbb {N}}_0$, $\varepsilon ,\delta _1,\delta _2,\ldots ,\delta _{2n-1}\in {\mathbb {R}}$ it holds that

71 $$\begin{aligned}  &   \!\!\! \textbf{u}^{\varepsilon ,m,(\delta _1,\delta _2,\ldots ,\delta _{2n-1})}_{2n-1,N} = ( v^{n,-\varepsilon }_{nN^m,N}, v^{1,\delta _1-\varepsilon }_{nN^m,N}, v^{1,\delta _2-\varepsilon }_{nN^m,N}, v^{2,\delta _3-\varepsilon }_{nN^m,N}, v^{2,\delta _4-\varepsilon }_{nN^m,N}, \ldots , \nonumber \\  &   v^{n-1,\delta _{2n-3}-\varepsilon }_{nN^m,N}, v^{n-1,\delta _{2n-2}-\varepsilon }_{nN^m,N}, N^m \, v^{n,\delta _{2n-1}-(\nicefrac {1}{2})-\varepsilon }_{nN^m,N} ). \end{aligned}$$

Moreover, observe that items (i)–(iv) of Lemma 2.2 establish items (i)–(iv). It thus remains to prove item (v). For this let $X^{n,\textbf{u}}:[0,T]\times \Omega \rightarrow H$, $\textbf{u}\in H^{n+1}$, $n\in {\mathbb {N}}_0$, be /-predictable stochastic processes such that for all $p\in [2,\infty )$, $n\in {\mathbb {N}}$, $\textbf{u}\in H^n$, $x\in H$, $t\in [0,T]$ it holds

(I) that $$ \sup _{s\in [0,T]} {\mathbb {E}}\big [\Vert X^{0,x}_s\Vert ^p_H\big ] < \infty $$,
(II) that
  72 $$\begin{aligned} \begin{aligned}&|\![X_t^{0,x} - e^{tA} x]\!| = \int _0^t e^{ ( t - s ) A } B(X_s^{0,x}) \, dW_s , \end{aligned} \end{aligned}$$
  and
(III) that
  73 $$\begin{aligned} \begin{aligned}&\big ( \tfrac{d^n}{dx^n} |\![X^{0,x}_t]\!| \big ) \textbf{u} = \big ( H \ni y \mapsto |\![X^{0,y}_t]\!| \in L^{p}({\mathbb {P}};H) \big )^{(n)}(x) \, \textbf{u} = |\![X^{n,(x,\textbf{u})}_t]\!| . \end{aligned} \end{aligned}$$

Next observe that items (i) and (iii) of Lemma 2.1 ensure that for all $n \in {\mathbb {N}}$, $r\in [0,\infty )$, $u_0, u_1, \ldots , u_n \in H$, $t\in [0,T]$ it holds that

74 $$\begin{aligned} \int ^t_0 \Vert e^{(t-s)A } B^{(n)}( e^{sA} u_0 ) ( e^{sA} u_1, e^{sA} u_2, \ldots , e^{sA} u_n ) \Vert ^2_{H_{-r}} \, ds < \infty . \end{aligned}$$

Item (v) of Lemma 2.2 and Itô’s isometry hence show that for all $n \in {\mathbb {N}}$, $r\in [0,\infty )$, $\textbf{u}= (u_0, u_1, \ldots , u_n) \in H^{n+1}$, $t\in [0,T]$ it holds that

75 $$\begin{aligned} \begin{aligned}&{\mathbb {E}}\big [ \Vert X^{ n,\textbf{u} }_t \Vert ^2_{H_{-r}} \big ] \\&\quad = \mathbb {1}_{\{1\}}(n) \, \Vert e^{tA} u_1 \Vert ^2_{H_{-r}} \\  &\qquad + \int ^t_0 \Vert (-A)^{-r} e^{(t-s)A } B^{(n)}( e^{sA} \theta ^{n+1}_{1}(\textbf{u}) ) ( e^{sA} \theta ^{n+1}_{2}(\textbf{u}), e^{sA} \theta ^{n+1}_{3}(\textbf{u}), \ldots , e^{sA} \theta ^{n+1}_{n+1}(\textbf{u}) ) \Vert ^2_H \,ds \\  &\quad \ge \int ^t_0 \Vert e^{(t-s)A } B^{(n)}( e^{sA} \theta ^{n+1}_{1}(\textbf{u}) ) ( e^{sA} \theta ^{n+1}_{2}(\textbf{u}), e^{sA} \theta ^{n+1}_{3}(\textbf{u}), \ldots , e^{sA} \theta ^{n+1}_{n+1}(\textbf{u}) ) \Vert ^2_{H_{-r}} \,ds . \end{aligned} \end{aligned}$$

In particular, this shows that for all $N,n \in {\mathbb {N}}$, $m \in {\mathbb {N}}_0$, $r\in [0,\infty )$, $\varepsilon ,\delta _1,\delta _2,\ldots ,\delta _{2n} \in {\mathbb {R}}$, $t \in [0,T]$ it holds that

76 $$\begin{aligned}  &   {\mathbb {E}}\Big [\big \Vert X^{ 2n-1,\textbf{u}^{\varepsilon ,m,(\delta _1,\delta _2,\ldots ,\delta _{2n-1})}_{2n-1,N} }_t \big \Vert ^2_{H_{-r}}\Big ] \nonumber \\  &   \quad \ge \int ^t_0 \big \Vert e^{(t-s)A } B^{(2n-1)}( e^{sA} \theta ^{2n}_1(\textbf{u}^{\varepsilon ,m,(\delta _1,\delta _2,\ldots ,\delta _{2n-1})}_{2n-1,N}) ) \big ( e^{sA} \theta ^{2n}_2(\textbf{u}^{\varepsilon ,m,(\delta _1,\delta _2,\ldots ,\delta _{2n-1})}_{2n-1,N}), \nonumber \\  &   e^{sA} \theta ^{2n}_3(\textbf{u}^{\varepsilon ,m,(\delta _1,\delta _2,\ldots ,\delta _{2n-1})}_{2n-1,N}), \ldots , e^{sA}\theta ^{2n}_{2n}(\textbf{u}^{\varepsilon ,m,(\delta _1,\delta _2,\ldots ,\delta _{2n-1})}_{2n-1,N}) \big ) \big \Vert ^2_{H_{-r}} \,ds \nonumber \\  &   \quad = \int ^t_0 \big \Vert e^{(t-s)A } \textbf{B}_{2n-1}\big (s,\textbf{u}^{\varepsilon ,m,(\delta _1,\delta _2,\ldots ,\delta _{2n-1})}_{2n-1,N}\big ) \big \Vert ^2_{H_{-r}} \,ds \end{aligned}$$

and

77 $$\begin{aligned}  &   {\mathbb {E}}\Big [\big \Vert X^{ 2n,\textbf{u}^{\varepsilon ,m,(\delta _1,\delta _2,\ldots ,\delta _{2n})}_{2n,N} }_t \big \Vert ^2_{H_{-r}}\Big ] \nonumber \\  &   \quad \ge \int ^t_0 \big \Vert e^{(t-s)A } B^{(2n)}( e^{sA} \theta ^{2n+1}_1(\textbf{u}^{\varepsilon ,m,(\delta _1,\delta _2,\ldots ,\delta _{2n})}_{2n,N}) ) \big ( e^{sA} \theta ^{2n+1}_2(\textbf{u}^{\varepsilon ,m,(\delta _1,\delta _2,\ldots ,\delta _{2n})}_{2n,N}), \nonumber \\  &   e^{sA} \theta ^{2n+1}_3(\textbf{u}^{\varepsilon ,m,(\delta _1,\delta _2,\ldots ,\delta _{2n})}_{2n,N}), \ldots , e^{sA}\theta ^{2n+1}_{2n+1}(\textbf{u}^{\varepsilon ,m,(\delta _1,\delta _2,\ldots ,\delta _{2n})}_{2n,N}) \big ) \big \Vert ^2_{H_{-r}} \,ds \nonumber \\  &   \quad = \int ^t_0 \big \Vert e^{(t-s)A } \textbf{B}_{2n}\big (s,\textbf{u}^{\varepsilon ,m,(\delta _1,\delta _2,\ldots ,\delta _{2n-1})}_{2n-1,N}\big ) \big \Vert ^2_{H_{-r}} \,ds. \end{aligned}$$

In the next steps we will estimate the right-hand sides of (76) and (77) to establish (92) and (100), respectively. We then combine (92) and (100) to establish (101). To this end we first consider the integrands on the right-hand sides of (76) and (77). As shown by item (ii) of Lemma 2.1 above, they have complex expressions containing summations over partitions of $\{1,2,\ldots ,n\}$, $n\in {\mathbb {N}}$. In order to simplify the summations we use an orthogonality property exhibited by the vectors $v^{ k, r }_{ n,N } \in H$, $N,n \in {\mathbb {N}}$, $k \in {\mathbb {N}}_0$, $r \in {\mathbb {R}}$, in (64). To be more specific, note that for all $N,n \in {\mathbb {N}}$, $k_1, k_2 \in \{1,2,\ldots ,n\}$, $r_1,r_2 \in {\mathbb {R}}$, $t \in [0,T]$ it holds that

78 $$\begin{aligned} \begin{aligned}&\langle P e^{tA} v^{k_1,r_1}_{n,N}, e^{tA} v^{k_2,r_2}_{n,N} \rangle _H = \langle P e^{tA} (-A)^{r_1} v^{k_1,0}_{n,N}, P e^{tA} (-A)^{r_2} v^{k_2,0}_{n,N} \rangle _H \\  &\quad = {\mathbb {1}}_{\{k_1\}}(k_2) \, \Vert P e^{tA} (-A)^{\nicefrac {(r_1+r_2)}{2}} v^{k_1,0}_{n,N} \Vert ^2_H = {\mathbb {1}}_{\{k_1\}}(k_2) \, \Vert P e^{tA} v^{k_1,\nicefrac {(r_1+r_2)}{2}}_{n,N} \Vert ^2_H . \end{aligned} \end{aligned}$$

In particular, this implies that for all $N,n \in {\mathbb {N}}$, $k_1, k_2 \in \{1,2,\ldots ,n\}$, $r_1,r_2 \in {\mathbb {R}}$, $t \in [0,T]$ with $k_1\ne k_2$ it holds that

79 $$\begin{aligned} \begin{aligned} \langle P e^{tA} v^{k_1,r_1}_{n,N}, e^{tA} v^{k_2,r_2}_{n,N} \rangle _H = 0 . \end{aligned} \end{aligned}$$

Equality (79) is an orthogonality property exhibited by the vectors $v^{ k, r }_{ n,N } \in H$, $N,n \in {\mathbb {N}}$, $k \in {\mathbb {N}}_0$, $r \in {\mathbb {R}}$, in (64). We will use this property to simplify the expressions of the integrands in the right-hand sides of (76) and (77) (see (82) and (96) below). We now proceed with estimating the right-hand side of (76). To this end we first observe that (71) implies that for all $N, n \in {\mathbb {N}}$, $m \in {\mathbb {N}}_0$, $\varepsilon , \delta _1,\delta _2, \ldots ,\delta _{2n-1} \in {\mathbb {R}}$, $t \in [0,T]$ it holds that

80 $$\begin{aligned} \begin{aligned}&\textbf{B}_{2n-1}\big (t,\textbf{u}^{\varepsilon ,m,(\delta _1,\delta _2,\ldots ,\delta _{2n-1})}_{2n-1,N}\big ) \\&\quad = B^{(2n-1)}( e^{tA} v^{n,-\varepsilon }_{nN^m,N} ) \big ( e^{tA} v^{1,\delta _1-\varepsilon }_{nN^m,N}, e^{tA} v^{1,\delta _2-\varepsilon }_{nN^m,N}, e^{tA} v^{2,\delta _3-\varepsilon }_{nN^m,N}, e^{tA} v^{2,\delta _4-\varepsilon }_{nN^m,N}, \\  &\qquad \ldots , e^{tA} v^{n-1,\delta _{2n-3}-\varepsilon }_{nN^m,N}, e^{tA} v^{n-1,\delta _{2n-2}-\varepsilon }_{nN^m,N}, N^m \, e^{tA} v^{n,\delta _{2n-1}-(\nicefrac {1}{2})-\varepsilon }_{nN^m,N} \big ) . \end{aligned} \end{aligned}$$

Item (ii) of Lemma 2.1 therefore yields that for all $N, n \in {\mathbb {N}}$, $m \in {\mathbb {N}}_0$, $\varepsilon , \delta _1,\delta _2, \ldots ,\delta _{2n-1} \in {\mathbb {R}}$, $t \in [0,T]$ it holds that

81 $$\begin{aligned}&\textbf{B}_{2n-1}\big (t,\textbf{u}^{\varepsilon ,m,(\delta _1,\delta _2,\ldots ,\delta _{2n-1})}_{2n-1,N}\big )\nonumber \\  &\quad = \Bigg ( {\sum \limits _{ \varpi \in \Pi _{2n-1} }} \Bigg ( \frac{ \big ( {\prod ^{ \#_\varpi - 1 }_{ i=0 }} (1-2i) \big ) }{ \big [ 1 + \Vert P e^{tA} v^{n,-\varepsilon }_{nN^m,N} \Vert ^2_H \big ]^{ ( \#_\varpi - \nicefrac {1}{2} ) } }\nonumber \\  &\quad \cdot \Bigg [ \prod _{ I \in \varpi } \bigg ( \Big< \mathbb {1}_{\{1\}}( \#_I ) \, P e^{tA} v^{n,-\varepsilon }_{nN^m,N} , N^{m\mathbb {1}_{\{2n-1\}}(\min (I))} e^{tA} v^{\lceil \nicefrac {\min (I)}{2} \rceil ,\delta _{\min (I)}-(\nicefrac {1}{2})\mathbb {1}_{\{2n-1\}}(\min (I))-\varepsilon }_{nN^m,N} \Big>_H\nonumber \\  &\qquad + \Big < \mathbb {1}_{\{2\}}( \#_I ) \, P \big ( N^{m\mathbb {1}_{\{2n-1\}}(\max (I))} e^{tA} v^{\lceil \nicefrac {\max (I)}{2} \rceil ,\delta _{\max (I)}-(\nicefrac {1}{2})\mathbb {1}_{\{2n-1\}}(\max (I))-\varepsilon }_{nN^m,N} \big ) ,\nonumber \\  &\quad \times e^{tA} v^{\lceil \nicefrac {\min (I)}{2} \rceil ,\delta _{\min (I)}-\varepsilon }_{nN^m,N} \Big >_H \bigg ) \Bigg ] \Bigg ) \Bigg ) e_1 . \end{aligned}$$

This and (79) imply that for all $N, n \in {\mathbb {N}}$, $m \in {\mathbb {N}}_0$, $\varepsilon , \delta _1,\delta _2, \ldots ,\delta _{2n-1} \in {\mathbb {R}}$, $t \in [0,T]$ it holds that

82 $$\begin{aligned} \begin{aligned}&\textbf{B}_{2n-1}\big (t,\textbf{u}^{\varepsilon ,m,(\delta _1,\delta _2,\ldots ,\delta _{2n-1})}_{2n-1,N}\big ) \\  &\quad = \Bigg ( \sum _{ \varpi \in \Pi _{2n-1}, \, \varpi = \{ \{1,2\}, \{3,4\}, \ldots , \{2n-3,2n-2\}, \{2n-1\} \} } \Bigg ( \frac{ \big (\! \prod ^{ \#_\varpi - 1 }_{ i=0 } (1-2i) \big ) }{ [ 1 + \Vert P e^{tA} v^{n,-\varepsilon }_{nN^m,N} \Vert ^2_H ]^{ ( \#_\varpi - 1/2 ) } } \\  &\qquad \cdot \big< P e^{tA} v^{n,-\varepsilon }_{nN^m,N} , N^m e^{tA} v^{n,\delta _{2n-1}-(\nicefrac {1}{2})-\varepsilon }_{nN^m,N} \big>_H \Bigg [ \prod ^{n-1}_{ i=1 } \big < P e^{tA} v^{i,\delta _{2i}-\varepsilon }_{nN^m,N} , e^{tA} v^{i,\delta _{2i-1}-\varepsilon }_{nN^m,N} \big >_H \Bigg ] \Bigg ) \Bigg ) e_1 . \end{aligned} \end{aligned}$$

Combining (78) and (82) shows that for all $N, n \in {\mathbb {N}}$, $m \in {\mathbb {N}}_0$, $\varepsilon , \delta _1,\delta _2, \ldots ,\delta _{2n-1} \in {\mathbb {R}}$, $t \in [0,T]$ it holds that

83 $$\begin{aligned} \textbf{B}_{2n-1}\big (t,\textbf{u}^{\varepsilon ,m,(\delta _1,\delta _2,\ldots ,\delta _{2n-1})}_{2n-1,N}\big )&= N^m \, \Bigg ( \frac{ \big (\! \prod ^{n-1}_{ i=0 } (1-2i) \big ) }{ [ 1 + \Vert P e^{tA} v^{n,-\varepsilon }_{nN^m,N} \Vert ^2_H ]^{ ( n - 1/2 ) } }\, \nonumber \\&\quad \Vert P e^{tA} v^{n,(\nicefrac {\delta _{(2n-1)}}{2})-(\nicefrac {1}{4})-\varepsilon }_{nN^m,N} \Vert ^2_H\nonumber \\  &\quad \cdot \Bigg [ \prod ^{n-1}_{i=1} \Vert P e^{tA} v^{i,(\nicefrac {(\delta _{2i-1}+\delta _{2i})}{2})-\varepsilon }_{nN^m,N} \Vert ^2_H \Bigg ] \Bigg ) e_1 . \end{aligned}$$

Hence, we obtain that for all $N,n \in {\mathbb {N}}$, $m \in {\mathbb {N}}_0$, $r\in [0,\infty )$, $\delta _1,\delta _2,\ldots ,\delta _{2n-1} \in {\mathbb {R}}$, $\varepsilon \in (0,\infty )$, $t \in [0,T]$, $s \in [0,t]$ it holds that

84 $$\begin{aligned} \begin{aligned}&\big \Vert e^{(t-s)A } \textbf{B}_{2n-1}\big (s,\textbf{u}^{\varepsilon ,m,(\delta _1,\delta _2,\ldots ,\delta _{2n-1})}_{2n-1,N}\big ) \big \Vert ^2_{H_{-r}} \\  &\quad = \bigg [ \frac{N^m}{c^r} \bigg ]^2 e^{ -2c(t-s) } \Bigg [ \frac{ \big ( \prod \nolimits ^{n-1}_{ i=0 } (1-2i) \big ) }{ [ 1 + \Vert P e^{sA} v^{n,-\varepsilon }_{nN^m,N} \Vert ^2_H ]^{ ( n - 1/2 ) } } \, \Vert P e^{sA} v^{n,(\nicefrac {\delta _{(2n-1)}}{2})-(\nicefrac {1}{4})-\varepsilon }_{nN^m,N} \Vert ^2_H \\  &\qquad \cdot \Bigg ( \prod ^{n-1}_{i=1} \Vert P e^{sA} v^{i,(\nicefrac {(\delta _{2i-1}+\delta _{2i})}{2})-\varepsilon }_{nN^m,N} \Vert ^2_H \Bigg ) \Bigg ]^2 . \end{aligned} \end{aligned}$$

Putting this into the right-hand side of (76) yields that for all $N,n \in {\mathbb {N}}$, $m \in {\mathbb {N}}_0$, $r\in [0,\infty )$, $\delta _1,\delta _2,\ldots ,\delta _{2n-1} \in {\mathbb {R}}$, $\varepsilon \in (0,\infty )$, $t \in (0,T]$ it holds that

85 $$\begin{aligned}&{\mathbb {E}}\Big [\big \Vert X^{ 2n-1,\textbf{u}^{\varepsilon ,m,(\delta _1,\delta _2,\ldots ,\delta _{2n-1})}_{2n-1,N} }_t \big \Vert ^2_{H_{-r}}\Big ] \ge \left[ \frac{ N^m \big ( \prod \nolimits ^{n-1}_{ i=0 } (1-2i) \big ) }{ c^r e^{ct} [ 1 + \sup _{s\in [0,T]}\Vert P e^{sA} v^{n,-\varepsilon }_{nN^m,N} \Vert ^2_H ]^{ ( n - 1/2 ) } } \right] ^2\nonumber \\  &\quad \cdot \int ^t_0 \Bigg [ \Vert P e^{sA} v^{n,(\nicefrac {\delta _{(2n-1)}}{2})-(\nicefrac {1}{4})-\varepsilon }_{nN^m,N} \Vert ^2_H \Bigg ( \prod ^{n-1}_{i=1} \Vert P e^{sA} v^{i,(\nicefrac {(\delta _{2i-1}+\delta _{2i})}{2})-\varepsilon }_{nN^m,N} \Vert ^2_H \Bigg ) \Bigg ]^2 \,ds . \end{aligned}$$

In the following steps we further estimate the integrands on the right-hand side of (85). Observe that the fact that $\forall \, n, j, k, l_1, l_2, \ldots , l_n \in {\mathbb {N}}, \, i \in \{1,2,\ldots ,n\} :l_i + jk \ge 2$ implies that for all $N, n, k, l_1, l_2, \ldots , l_n \in {\mathbb {N}}$, $r_1, r_2, \ldots , r_n \in {\mathbb {R}}$, $t \in [0,T]$ it holds that

86 $$\begin{aligned}&\prod ^n_{i=1} \big \Vert P e^{tA} v^{l_i,r_i}_{k,N} \big \Vert ^2_H = \prod ^n_{i=1} \big \Vert P e^{tA}(-A)^{r_i} {\textstyle \sum ^N_{j=1}} e_{l_i+jk} \big \Vert ^2_H = \prod ^n_{i=1} \big \Vert e^{tA}(-A)^{r_i}\nonumber \\  &{\textstyle \sum ^N_{j=1}} e_{l_i+jk} \big \Vert ^2_H. \end{aligned}$$

This shows that for all $N, n, k, l_1, l_2, \ldots , l_n \in {\mathbb {N}}$, $r_1, r_2, \ldots , r_n \in {\mathbb {R}}$, $t \in [0,T]$ it holds that

87 $$\begin{aligned} \begin{aligned}&\prod ^n_{i=1} \big \Vert P e^{tA} v^{l_i,r_i}_{k,N} \big \Vert ^2_H = \prod ^n_{i=1} \left( \sum ^N_{j=1} \left[ e^{-c(l_i+jk)^2 t} \, \Vert (-A)^{r_i} e_{l_i+jk}\Vert _H \right] ^2 \right) \\  &\quad = \sum ^N_{j_1=1} \sum ^N_{j_2=1} \ldots \sum ^N_{j_n=1} \left( \prod ^n_{i=1} \left[ e^{-c(l_i+j_i k)^2 t} \, \Vert (-A)^{r_i} e_{l_i+j_i k}\Vert _H \right] ^2 \right) \\  &\quad \ge \sum ^N_{j_1=1} \sum ^{j_1}_{j_2=j_1} \sum ^{j_1}_{j_3=j_1} \ldots \sum ^{j_1}_{j_n=j_1} \left( \prod ^n_{i=1} \left[ e^{-c(l_i+j_i k)^2 t} \, \Vert (-A)^{r_i} e_{l_i+j_i k}\Vert _H \right] ^2 \right) \\  &\quad = \sum ^N_{j=1} \left( \prod ^n_{i=1} \left[ e^{-c(l_i+jk)^2 t} \, \Vert (-A)^{r_i} e_{l_i+jk}\Vert _H \right] ^2 \right) = \sum ^N_{j=1} \left( \prod ^n_{i=1} \left[ e^{-c(l_i+jk)^2 t} \, [c\,(l_i+jk)^2]^{r_i} \right] ^2 \right) . \end{aligned} \end{aligned}$$

This and the fact that

88 $$\begin{aligned} \forall \, x \in {\mathbb {R}}:x=\max \{x,0\}+\min \{x,0\}=\max \{x,0\}-\max \{-x,0\} \end{aligned}$$

ensure that for all $N, n, k, l_1, l_2, \ldots , l_n \in {\mathbb {N}}$, $r_1, r_2, \ldots , r_n \in {\mathbb {R}}$, $t \in [0,T]$ it holds that

89 $$\begin{aligned} \begin{aligned} \prod ^n_{i=1} \big \Vert P e^{tA} v^{l_i,r_i}_{k,N} \big \Vert ^2_H&\ge \sum ^N_{j=1} \left( \prod ^n_{i=1} \Bigg [ \big | e^{-c(l_i+jk)^2 t} \, [c\,(\nicefrac {jk}{n})^2]^{r_i} \big |^2 \bigg [ \frac{l_i+jk}{(\nicefrac {jk}{n})} \bigg ]^{4r_i} \Bigg ] \right) \\  &\ge \sum ^N_{j=1} \left( \prod ^n_{i=1} \Bigg [ \frac{ \big | e^{-c(l_i+jk)^2 t} \, [c\,(\nicefrac {jk}{n})^2]^{r_i} \big |^2 }{ ( n+\nicefrac {nl_i}{(jk)} )^{4\max \{-r_i,0\}} } \Bigg ] \right) \\  &\ge \sum ^N_{j=1} \left( \prod ^n_{i=1} \Bigg [ \frac{ \big | e^{-c(l_i+jk)^2 t} \, [c\,(\nicefrac {jk}{n})^2]^{r_i} \big |^2 }{ ( n+n\max _{m\in \{1,2,\ldots ,n\}} l_m )^{4\max \{-r_i,0\}} } \Bigg ] \right) . \end{aligned} \end{aligned}$$

This assures that for all $N, n, l_1, l_2, \ldots , l_n \in {\mathbb {N}}$, $k \in \{n,2n,3n,\ldots \}$, $r_1, r_2, \ldots , r_n \in {\mathbb {R}}$, $t \in [0,T]$ it holds that

90 $$\begin{aligned} \begin{aligned}&\prod ^n_{i=1} \big \Vert P e^{tA} v^{l_i,r_i}_{k,N} \big \Vert ^2_H \ge \frac{1}{ ( n+n\max _{i\in \{1,2,\ldots ,n\}} l_i )^{4\sum ^n_{i=1}|r_i|} } \left[ \sum ^N_{j=1} \left( \prod ^n_{i=1} \big | e^{-c(l_i+jk)^2 t} \, [c\,(\nicefrac {jk}{n})^2]^{r_i} \big |^2 \right) \right] \\  &\quad \ge \frac{1}{ ( n+n\max _{i\in \{1,2,\ldots ,n\}} l_i )^{4\sum ^n_{i=1}|r_i|} } \left[ \sum ^N_{j=1} \left( \prod ^n_{i=1} \big | e^{-2c(l_i)^2 t} \, e^{-2c(jk)^2 t} \, [c\,(\nicefrac {jk}{n})^2]^{r_i} \big |^2 \right) \right] \\  &\quad = \frac{ e^{-4ct\sum ^n_{i=1} |l_i|^2} }{ ( n+n\max _{i\in \{1,2,\ldots ,n\}} l_i )^{4\sum ^n_{i=1}|r_i|} } \left[ \sum ^N_{j=1} \big | e^{-2c(jk)^2 n t} \, [c\,(\nicefrac {jk}{n})^2]^{\sum ^n_{i=1} r_i} \big |^2 \right] . \end{aligned} \end{aligned}$$

Therefore, we obtain that for all $N, n, l_1, l_2, \ldots , l_n \in {\mathbb {N}}$, $k \in \{n,2n,3n,\ldots \}$, $r_1, r_2, \ldots , r_n \in {\mathbb {R}}$, $t \in [0,T]$ it holds that

91 $$\begin{aligned} \begin{aligned} \prod ^n_{i=1} \big \Vert P e^{tA} v^{l_i,r_i}_{k,N} \big \Vert ^2_H&\ge \frac{ e^{-4ctn\max _{i\in \{1,2,\ldots ,n\}} |l_i|^2} }{ ( n+n\max _{i\in \{1,2,\ldots ,n\}} l_i )^{4\sum ^n_{i=1}|r_i|} } \left[ \sum ^N_{j=1} \big | e^{-2n^3tc(\nicefrac {jk}{n})^2} \, [c\,(\nicefrac {jk}{n})^2]^{\sum ^n_{i=1} r_i} \big |^2 \right] \\  &= \frac{ e^{-4ctn\max _{i\in \{1,2,\ldots ,n\}} |l_i|^2} }{ ( n+n\max _{i\in \{1,2,\ldots ,n\}} l_i )^{4\sum ^n_{i=1}|r_i|} } \, \Vert e^{2n^3tA} \, v^{0,\sum ^n_{i=1}r_i}_{\nicefrac {k}{n},N}\Vert ^2_H . \end{aligned} \end{aligned}$$

Combining this with (85) ensures that for all $N,n \in {\mathbb {N}}$, $m \in {\mathbb {N}}_0$, $r\in [0,\infty )$, $\delta _1,\delta _2,\ldots ,\delta _{2n-1} \in {\mathbb {R}}$, $\varepsilon \in (0,\infty )$, $t \in (0,T]$ it holds that

92 $$\begin{aligned} \begin{aligned}&{\mathbb {E}}\Big [\big \Vert X^{ 2n-1,\textbf{u}^{\varepsilon ,m,(\delta _1,\delta _2,\ldots ,\delta _{2n-1})}_{2n-1,N} }_t \big \Vert ^2_{H_{-r}}\Big ] \\  &\quad \ge \left[ \frac{ N^m \big ( \prod \nolimits ^{n-1}_{ i=0 } (1-2i) \big ) }{ c^r e^{ct} [ 1 + \sup _{s\in [0,T]}\Vert P e^{sA} v^{n,-\varepsilon }_{nN^m,N} \Vert ^2_H ]^{ ( n - 1/2 ) } } \right] ^2 \\  &\qquad \cdot \int ^t_0 \frac{ e^{ -8cn^3s } \Vert e^{2n^3sA} v^{0,-(\nicefrac {1}{4})-n\varepsilon +\sum ^{2n-1}_{i=1}(\nicefrac {\delta _i}{2})}_{N^m,N} \Vert ^4_H }{ (n+n^2)^{8(|(\nicefrac {\delta _{(2n-1)}}{2})-(\nicefrac {1}{4})-\varepsilon |+\sum ^{n-1}_{i=1}|(\nicefrac {(\delta _{2i-1}+\delta _{2i})}{2})-\varepsilon |)} } \,ds \\  &\quad \ge \left[ \frac{ N^m \big ( \prod \nolimits ^{n-1}_{ i=0 } (1-2i) \big ) }{ c^r e^{ c(1+4n^3) t} (2n^2)^{(1+4n\varepsilon +2\sum ^{2n-1}_{i=1}|\delta _i|)} \, [ 1 + \sup _{s\in [0,T]} \Vert P e^{sA} v^{n,-\varepsilon }_{nN^m,N} \Vert ^2_H ]^{ ( n - 1/2 ) } } \right] ^2 \\  &\qquad \cdot \int ^t_0 \Vert e^{2n^3sA} v^{0,-(\nicefrac {1}{4})-n\varepsilon +\sum ^{2n-1}_{i=1}(\nicefrac {\delta _i}{2})}_{N^m,N} \Vert ^4_H \,ds . \end{aligned} \end{aligned}$$

We next estimate the right-hand side of (77). To this end we note that (66) shows that for all $N, n \in {\mathbb {N}}$, $m \in {\mathbb {N}}_0$, $\varepsilon , \delta _1,\delta _2, \ldots ,\delta _{2n} \in {\mathbb {R}}$, $t \in [0,T]$ it holds that

93 $$\begin{aligned} \begin{aligned}&\textbf{B}_{2n}\big (t,\textbf{u}^{\varepsilon ,m,(\delta _1,\delta _2,\ldots ,\delta _{2n})}_{2n,N}\big ) \\  &\quad = B^{(2n)}( e^{tA} e_1 ) \big ( e^{tA} v^{1,\delta _1-\varepsilon }_{nN^m,N}, e^{tA} v^{1,\delta _2-\varepsilon }_{nN^m,N}, e^{tA} v^{2,\delta _3-\varepsilon }_{nN^m,N}, e^{tA} v^{2,\delta _4-\varepsilon }_{nN^m,N}, \\  &\qquad \ldots , e^{tA} v^{n-1,\delta _{2n-3}-\varepsilon }_{nN^m,N}, e^{tA} v^{n-1,\delta _{2n-2}-\varepsilon }_{nN^m,N}, e^{tA} v^{n,\delta _{2n-1}-\varepsilon }_{nN^m,N}, N^m \, e^{tA} v^{n,\delta _{2n}-(\nicefrac {1}{2})-\varepsilon }_{nN^m,N} \big ) . \end{aligned} \end{aligned}$$

Item (ii) of Lemma 2.1 therefore ensures that for all $N, n \in {\mathbb {N}}$, $m \in {\mathbb {N}}_0$, $\varepsilon , \delta _1,\delta _2, \ldots ,\delta _{2n} \in {\mathbb {R}}$, $t \in [0,T]$ it holds that

94 $$\begin{aligned} \begin{aligned}&\textbf{B}_{2n}\big (t,\textbf{u}^{\varepsilon ,m,(\delta _1,\delta _2,\ldots ,\delta _{2n})}_{2n,N}\big ) \\  &\quad = \Bigg ( {\sum \limits _{ \varpi \in \Pi _{2n} }} \Bigg ( \frac{ \big ( {\prod ^{ \#_\varpi - 1 }_{ i=0 }} (1-2i) \big ) }{ \big [ 1 + \Vert P e^{tA} e_1 \Vert ^2_H \big ]^{ ( \#_\varpi - \nicefrac {1}{2} ) } } \\  &\qquad \cdot \Bigg [ \prod _{ I \in \varpi } \bigg ( \Big< \mathbb {1}_{\{1\}}( \#_I ) \, P e^{tA} e_1 , N^{m\mathbb {1}_{\{2n\}}(\min (I))} e^{tA} v^{\lceil \nicefrac {\min (I)}{2} \rceil ,\delta _{\min (I)}-(\nicefrac {1}{2})\mathbb {1}_{\{2n\}}(\min (I))-\varepsilon }_{nN^m,N} \Big>_H \\  &\qquad + \Big < \mathbb {1}_{\{2\}}( \#_I ) \, P \big ( N^{m\mathbb {1}_{\{2n\}}(\max (I))} e^{tA} v^{\lceil \nicefrac {\max (I)}{2} \rceil ,\delta _{\max (I)}-(\nicefrac {1}{2})\mathbb {1}_{\{2n\}}(\max (I))-\varepsilon }_{nN^m,N} \big ) ,\\  &\qquad e^{tA} v^{\lceil \nicefrac {\min (I)}{2} \rceil ,\delta _{\min (I)}-\varepsilon }_{nN^m,N} \Big >_H \bigg ) \Bigg ] \Bigg ) \Bigg ) e_1 . \end{aligned} \end{aligned}$$

Next observe that the fact that $P e_1 = 0$ ensures that for all $t\in [0,T]$ it holds that $P e^{tA} e_1 = 0$. This and (94) show that for all $N, n \in {\mathbb {N}}$, $m \in {\mathbb {N}}_0$, $\varepsilon , \delta _1,\delta _2, \ldots ,\delta _{2n} \in {\mathbb {R}}$, $t \in [0,T]$ it holds that

95 $$\begin{aligned} \begin{aligned}&\textbf{B}_{2n}\big (t,\textbf{u}^{\varepsilon ,m,(\delta _1,\delta _2,\ldots ,\delta _{2n})}_{2n,N}\big ) \\  &\quad = \Bigg ( {\sum \limits _{ \begin{array}{c} \varpi \in \Pi _{2n}, \, \forall \, I \in \varpi :\#_I = 2 \end{array} }} \Bigg ( \Bigg [ {\prod ^{ \#_\varpi - 1 }_{ i=0 }} (1-2i) \Bigg ] \\  &\qquad \cdot \Bigg [ \prod _{\begin{array}{c} I \in \varpi \end{array}} \Big < P \big ( N^{m\mathbb {1}_{\{2n\}}(\max (I))} e^{tA} v^{\lceil \nicefrac {\max (I)}{2} \rceil ,\delta _{\max (I)}-(\nicefrac {1}{2})\mathbb {1}_{\{2n\}}(\max (I))-\varepsilon }_{nN^m,N} \big ) ,\\  &\quad e^{tA} v^{\lceil \nicefrac {\min (I)}{2} \rceil ,\delta _{\min (I)}-\varepsilon }_{nN^m,N} \Big >_H \Bigg ] \Bigg ) \Bigg ) e_1 . \end{aligned} \end{aligned}$$

This and (79) assure that for all $N, n \in {\mathbb {N}}$, $m \in {\mathbb {N}}_0$, $\varepsilon , \delta _1,\delta _2, \ldots ,\delta _{2n} \in {\mathbb {R}}$, $t \in [0,T]$ it holds that

96 $$\begin{aligned} \begin{aligned}&\textbf{B}_{2n}\big (t,\textbf{u}^{\varepsilon ,m,(\delta _1,\delta _2,\ldots ,\delta _{2n})}_{2n,N}\big ) \\  &\quad = \Bigg ( \sum _{ \varpi \in \Pi _{2n}, \, \varpi = \{ \{1,2\}, \{3,4\}, \ldots , \{2n-3,2n-2\}, \{2n-1,2n\} \} } \Bigg ( \Bigg [ {\prod \limits ^{ \#_\varpi - 1 }_{ i=0 } (1-2i)} \Bigg ] \\  &\qquad \cdot \big< P\big ( N^m e^{tA} v^{n,\delta _{2n}-(\nicefrac {1}{2})-\varepsilon }_{nN^m,N} \big ) , e^{tA} v^{n,\delta _{2n-1}-\varepsilon }_{nN^m,N} \big>_H \Bigg [ \prod ^{n-1}_{ i=1 } \big < P e^{tA} v^{i,\delta _{2i}-\varepsilon }_{nN^m,N} , e^{tA} v^{i,\delta _{2i-1}-\varepsilon }_{nN^m,N} \big >_H \Bigg ] \Bigg ) \Bigg ) \, e_1 . \end{aligned} \end{aligned}$$

Furthermore, observe that (78) implies that for all $N, n \in {\mathbb {N}}$, $m \in {\mathbb {N}}_0$, $\varepsilon , \delta _1,\delta _2, \ldots ,\delta _{2n} \in {\mathbb {R}}$, $t \in [0,T]$ it holds that

97 $$\begin{aligned}&\textbf{B}_{2n}\big (t,\textbf{u}^{\varepsilon ,m,(\delta _1,\delta _2,\ldots ,\delta _{2n})}_{2n,N}\big )\nonumber \\  &\quad = N^m \, \Bigg ( \left[ \prod ^{n-1}_{ i=0 } (1-2i) \right] \Vert P e^{tA} v^{n,(\nicefrac {(\delta _{2n-1}+\delta _{2n})}{2})-(\nicefrac {1}{4})-\varepsilon }_{nN^m,N} \Vert ^2_H \Bigg [ \prod ^{n-1}_{i=1} \Vert P e^{tA} v^{i,(\nicefrac {(\delta _{2i-1}+\delta _{2i})}{2})-\varepsilon }_{nN^m,N} \Vert ^2_H \Bigg ] \Bigg ) \, e_1. \end{aligned}$$

Therefore, we obtain that for all $N,n \in {\mathbb {N}}$, $m \in {\mathbb {N}}_0$, $r\in [0,\infty )$, $\delta _1,\delta _2,\ldots ,\delta _{2n} \in {\mathbb {R}}$, $\varepsilon \in (0,\infty )$, $t \in [0,T]$, $s \in [0,t]$ it holds that

98 $$\begin{aligned} \begin{aligned}&\big \Vert e^{(t-s)A } \textbf{B}_{2n}\big (s,\textbf{u}^{\varepsilon ,m,(\delta _1,\delta _2,\ldots ,\delta _{2n})}_{2n,N}\big ) \big \Vert ^2_{H_{-r}} \\  &\quad = \bigg [ \frac{N^m}{c^r} \bigg ]^2 e^{ -2c(t-s) } \Bigg [ \left( \prod ^{n-1}_{ i=0 } (1-2i) \right) \Vert P e^{sA} v^{n,\nicefrac {((\delta _{2n-1}+\delta _{2n})}{2})-(\nicefrac {1}{4})-\varepsilon }_{nN^m,N} \Vert ^2_H \\  &\qquad \cdot \Bigg ( \prod ^{n-1}_{i=1} \Vert P e^{sA} v^{i,\nicefrac {((\delta _{2i-1}+\delta _{2i})}{2})-\varepsilon }_{nN^m,N} \Vert ^2_H \Bigg )\Bigg ]^2 . \end{aligned} \end{aligned}$$

Putting this into the right-hand side of (77) yields that for all $N,n \in {\mathbb {N}}$, $m \in {\mathbb {N}}_0$, $r\in [0,\infty )$, $\delta _1,\delta _2,\ldots ,\delta _{2n} \in {\mathbb {R}}$, $\varepsilon \in (0,\infty )$, $t \in [0,T]$ it holds that

99 $$\begin{aligned} \begin{aligned}&{\mathbb {E}}\Big [\big \Vert X^{ 2n,\textbf{u}^{\varepsilon ,m,(\delta _1,\delta _2,\ldots ,\delta _{2n})}_{2n,N} }_t \big \Vert ^2_{H_{-r}}\Big ] \\  &\quad \ge \bigg [ \frac{N^m}{c^r} \bigg ]^2 \int ^t_0 e^{ -2c(t-s) } \Bigg [ \left( \prod ^{n-1}_{ i=0 } (1-2i) \right) \Vert P e^{sA} v^{n,\nicefrac {((\delta _{2n-1}+\delta _{2n})}{2})-(\nicefrac {1}{4})-\varepsilon }_{nN^m,N} \Vert ^2_H \\  &\qquad \cdot \Bigg ( \prod ^{n-1}_{i=1} \Vert P e^{sA} v^{i,\nicefrac {((\delta _{2i-1}+\delta _{2i})}{2})-\varepsilon }_{nN^m,N} \Vert ^2_H \Bigg )\Bigg ]^2 \,ds \\  &\quad \ge \left[ \frac{ N^m \big ( \prod \nolimits ^{n-1}_{ i=0 } (1-2i) \big ) }{ c^r e^{ ct } } \right] ^2 \\  &\quad \cdot \int ^t_0 \left[ \Vert P e^{sA} v^{n,\nicefrac {((\delta _{2n-1}+\delta _{2n})}{2})-(\nicefrac {1}{4})-\varepsilon }_{nN^m,N} \Vert ^2_H \Bigg ( \prod ^{n-1}_{i=1} \Vert P e^{sA} v^{i,(\nicefrac {(\delta _{2i-1}+\delta _{2i})}{2})-\varepsilon }_{nN^m,N} \Vert ^2_H \Bigg ) \right] ^2 \,ds . \end{aligned} \end{aligned}$$

This and (91) assure that for all $N,n \in {\mathbb {N}}$, $m \in {\mathbb {N}}_0$, $r\in [0,\infty )$, $\delta _1,\delta _2,\ldots ,\delta _{2n} \in {\mathbb {R}}$, $\varepsilon \in (0,\infty )$, $t \in [0,T]$ it holds that

100 $$\begin{aligned}&{\mathbb {E}}\Big [\big \Vert X^{ 2n,\textbf{u}^{\varepsilon ,m,(\delta _1,\delta _2,\ldots ,\delta _{2n})}_{2n,N} }_t \big \Vert ^2_{H_{-r}}\Big ]\nonumber \\  &\quad \ge \left[ \frac{ N^m \big ( \prod \nolimits ^{n-1}_{ i=0 } (1-2i) \big ) }{ c^r e^{ ct } } \right] ^2 \int ^t_0 \frac{ e^{-8cn^3s} \Vert e^{2n^3sA} v^{0,-(\nicefrac {1}{4})-n\varepsilon +\sum ^{2n}_{i=1}(\nicefrac {\delta _i}{2})}_{N^m,N} \Vert ^4_H }{ (n+n^2)^{8(|(\nicefrac {(\delta _{2n-1}+\delta _{2n})}{2})-(\nicefrac {1}{4})-\varepsilon |+\sum ^{n-1}_{i=1}|(\nicefrac {(\delta _{2i-1}+\delta _{2i})}{2})-\varepsilon |)} } \,ds\nonumber \\  &\quad \ge \left[ \frac{ N^m \big ( \prod \nolimits ^{n-1}_{ i=0 } (1-2i) \big ) }{ c^r e^{ c(1+4n^3)t } (2n^2)^{(1+4n\varepsilon +2\sum ^{2n}_{i=1}|\delta _i|)} } \right] ^2 \int ^t_0 \Vert e^{2n^3sA} v^{0,-(\nicefrac {1}{4})-n\varepsilon +\sum ^{2n}_{i=1}(\nicefrac {\delta _i}{2})}_{N^m,N} \Vert ^4_H \,ds . \end{aligned}$$

This and (92) yield that for all $N, n \in {\mathbb {N}}$, $r\in [0,\infty )$, $\delta _1,\delta _2,\ldots ,\delta _n \in {\mathbb {R}}$, $\varepsilon \in (0,\nicefrac {1}{4})$, $m\in {\mathbb {N}}_0\cap [\frac{1}{4\varepsilon }-1,\infty )$, $t \in (0,T]$ it holds that

101 $$\begin{aligned} \begin{aligned}&{\mathbb {E}}\Big [\big \Vert X^{ n,\textbf{u}^{\varepsilon ,m,(\delta _1,\delta _2,\ldots ,\delta _n)}_{n,N} }_t \big \Vert ^2_{H_{-r}}\Big ] \\  &\quad \ge \left[ \frac{ N^m \big ( \prod \nolimits ^{ \lceil \nicefrac {n}{2} \rceil - 1 }_{ i=0 } (1-2i) \big ) }{ c^r e^{ c(1+4|\lceil n/2 \rceil |^3) t} (2|\lceil n/2 \rceil |^2)^{(1+4\lceil n/2 \rceil \varepsilon +2\sum ^n_{i=1}|\delta _i|)} \, [ 1 + \sup _{s\in [0,T]} \Vert P e^{sA} v^{\lceil n/2 \rceil ,-\varepsilon }_{\lceil n/2 \rceil N^m,N} \Vert ^2_H ]^{ ( \lceil \nicefrac {n}{2} \rceil - \nicefrac {1}{2} ) } } \right] ^2 \\  &\qquad \cdot \int ^t_0 \big \Vert e^{2|\lceil \nicefrac {n}{2}\rceil |^3sA} v^{0,-(\nicefrac {1}{4})-\lceil \nicefrac {n}{2}\rceil \varepsilon +\sum ^n_{i=1}(\nicefrac {\delta _i}{2})}_{N^m,N} \big \Vert ^4_H \,ds . \end{aligned} \end{aligned}$$

In the next steps we estimate the two norms appearing in the right-hand side of (101) with the help of our choice of vectors $v^{ k, r }_{ n,N } \in H$, $N,n \in {\mathbb {N}}$, $k \in {\mathbb {N}}_0$, $r \in {\mathbb {R}}$, in (64) and the condition $\sum ^n_{i=1} \delta _i > \frac{1}{2}$ in order to establish (106). After that, the remaining steps of the proof is to establish (112) and (113) below. The last part of the proof (see inequality (113) below) is a direct consequence of (106) and (112). We now proceed to establish (106). To this end we first note that for all $N,n\in {\mathbb {N}}$, $i\in \{1,2,\ldots ,n\}$, $\varepsilon \in (0,\nicefrac {1}{4})$, $m\in {\mathbb {N}}_0\cap [\frac{1}{4\varepsilon }-1,\infty )$, $t\in [0,T]$ it holds that

102 $$\begin{aligned} \begin{aligned}&\Vert P e^{tA} v^{i,-\varepsilon }_{nN^m,N}\Vert ^2_H = \sum ^N_{j=1} \Vert e^{tA} (-A)^{-\varepsilon } e_{i+jnN^m} \Vert ^2_H \\  &\quad = \frac{1}{c^{2\varepsilon }} \left[ \sum ^N_{j=1} \frac{e^{-2tc(i+jnN^m)^2}}{ (i+jnN^m)^{4\varepsilon } } \right] \le \frac{1}{c^{2\varepsilon } N^{4m\varepsilon }} \left[ 1+ \sum ^N_{j=2} \frac{1}{ j^{4\varepsilon } } \right] \\  &\quad \le \frac{1}{c^{2\varepsilon } N^{4m\varepsilon }} \bigg ( 1+ \int ^N_1 \frac{1}{ x^{4\varepsilon } } \, dx \bigg ) = \frac{1}{c^{2\varepsilon }} \bigg ( \frac{1}{N^{4m\varepsilon }}+ \frac{1}{(1-4\varepsilon )} \bigg [ N^{(1-4\varepsilon -4m\varepsilon )}-\frac{1}{N^{4m\varepsilon }} \bigg ] \bigg ) . \end{aligned} \end{aligned}$$

This and the fact that $\forall \, \varepsilon \in (0,\nicefrac {1}{4}), \, m\in {\mathbb {N}}_0\cap [\frac{1}{4\varepsilon }-1,\infty ) :1-4\varepsilon -4\,m\varepsilon \le 0$ ensure that for all $N,n\in {\mathbb {N}}$, $i\in \{1,2,\ldots ,n\}$, $\varepsilon \in (0,\nicefrac {1}{4})$, $m\in {\mathbb {N}}_0\cap [\frac{1}{4\varepsilon }-1,\infty )$ it holds that

103 $$\begin{aligned} \begin{aligned} \sup _{t\in [0,T]} \Vert P e^{tA} v^{i,-\varepsilon }_{nN^m,N}\Vert ^2_H \le \frac{1}{c^{2\varepsilon }} \bigg ( \frac{1}{N^{4m\varepsilon }}+ \frac{1}{(1-4\varepsilon )}- \frac{1}{(1-4\varepsilon )N^{4m\varepsilon }} \bigg ) \le \frac{1}{c^{2\varepsilon }(1-4\varepsilon )} . \end{aligned} \end{aligned}$$

Next note that for all $N,l \in {\mathbb {N}}$, $m \in {\mathbb {N}}_0$, $\varepsilon \in (0,\infty )$, $\delta \in [\nicefrac {1}{2}+2\,l\varepsilon ,\infty )$, $t \in (0,T]$ it holds that

104 $$\begin{aligned} \begin{aligned}&\int ^t_0 \big \Vert e^{2l^3sA} v^{0,-(\nicefrac {1}{4})-l\varepsilon +(\nicefrac {\delta }{2})}_{N^m,N} \big \Vert ^4_H \,ds = \int _0^t \left[ {\sum _{ j = 1 }^N} \left( e^{ -2l^3cj^2N^{2m} s } \big \Vert (-A)^{-(\nicefrac {1}{4})-l\varepsilon +(\nicefrac {\delta }{2})} e_{jN^m} \big \Vert _H \right) ^2 \right] ^2 ds \\  &\quad = \sum _{ j, k = 1 }^N \Vert (-A)^{-(\nicefrac {1}{4})-l\varepsilon +(\nicefrac {\delta }{2})} e_{jN^m} \Vert ^2_H \, \Vert (-A)^{-(\nicefrac {1}{4})-l\varepsilon +(\nicefrac {\delta }{2})} e_{kN^m} \Vert ^2_H \left[ \int _0^t e^{ -4l^3c ( j^2 + k^2 )N^{2m} s } \, ds \right] \\  &\quad = \sum _{ j, k = 1 }^{ N } \left( \left[ \frac{ ( 1 - e^{ -4l^3c (j^2+k^2) N^{2m} t } ) }{ 4l^3c^{(2+4l\varepsilon -2\delta )} (j^2 + k^2) N^{2m} } \right] (jN^m)^{ ( 2\delta -1 -4l\varepsilon ) } \, (kN^m)^{ ( 2\delta -1 -4l\varepsilon ) } \right) . \end{aligned} \end{aligned}$$

The fact that $\forall \, l \in {\mathbb {N}}, \, \varepsilon \in (0,\infty ), \, \delta \in [\nicefrac {1}{2}+2\,l\varepsilon ,\infty ) :2\delta -1-4\,l\varepsilon \ge 0$ therefore assures that for all $N,l \in {\mathbb {N}}$, $m \in {\mathbb {N}}_0$, $\varepsilon \in (0,\infty )$, $\delta \in [\nicefrac {1}{2}+2\,l\varepsilon ,\infty )$, $t \in (0,T]$ it holds that

105 $$\begin{aligned} \begin{aligned} \int ^t_0 \big \Vert e^{2l^3sA} v^{0,-(\nicefrac {1}{4})-l\varepsilon +(\nicefrac {\delta }{2})}_{N^m,N} \big \Vert ^4_H \,ds&\ge \sum _{ j, k = 1 }^{ N } \frac{ ( 1 - e^{ -4l^3c (j^2+k^2) N^{2m} t } ) }{ 4l^3c^{(2+4l\varepsilon -2\delta )} (j^2 + k^2) N^{2m} } \\  &\ge \frac{ ( 1 - e^{ -ct } ) }{ 4l^3c^{(2+4l\varepsilon -2\delta )} N^{2m}} \left[ \sum _{ j, k = 1 }^N \frac{1}{ (j^2+k^2) } \right] . \end{aligned} \end{aligned}$$

Combining (101) with (103) and (105) yields that for all $n \in {\mathbb {N}}$, $r\in [0,\infty )$, $\delta _1,\delta _2,\ldots ,\delta _n \in {\mathbb {R}}$, $\varepsilon \in (0,\nicefrac {1}{4})$, $m\in {\mathbb {N}}_0\cap [\frac{1}{4\varepsilon }-1,\infty )$, $t \in (0,T]$ with $\sum ^n_{i=1} \delta _i \ge \frac{1}{2} + 2\lceil \nicefrac {n}{2}\rceil \varepsilon$ it holds that

106 $$\begin{aligned} \begin{aligned}&\sup _{N\in {\mathbb {N}}} \big ({\mathbb {E}}\big [\Vert X^{ n,\textbf{u}^{\varepsilon ,m,(\delta _1,\delta _2,\ldots ,\delta _n)}_{n,N} }_t \Vert ^2_{H_{-r}}\big ]\big )^{\nicefrac {1}{2}} \\  &\quad \ge \sup _{N\in {\mathbb {N}}}\bigg ( \frac{ N^m\prod \nolimits ^{ \lceil \nicefrac {n}{2} \rceil - 1 }_{ i=0 } |1-2i| }{ c^r e^{ c(1+4|\lceil n/2 \rceil |^3) t} (2|\lceil n/2 \rceil |^2)^{(1+4\lceil n/2 \rceil \varepsilon +2\sum ^n_{i=1}|\delta _i|)} [ 1 + \frac{1}{c^{2\varepsilon }(1-4\varepsilon )} ]^{ ( \lceil \nicefrac {n}{2} \rceil - \nicefrac {1}{2} ) } } \\  &\qquad \cdot \bigg [ \frac{ ( 1 - e^{ -ct } ) }{ 4|\lceil \nicefrac {n}{2}\rceil |^3c^{(2+4\lceil \nicefrac {n}{2}\rceil \varepsilon -2\sum ^n_{i=1}\delta _i)} N^{2m} } \sum _{ j, k = 1 }^N \frac{1}{ (j^2+k^2) } \bigg ]^{\nicefrac {1}{2}} \bigg ) \\  &\quad = \frac{ \prod \nolimits ^{ \lceil \nicefrac {n}{2} \rceil - 1 }_{ i=0 } |1-2i| }{ c^r e^{ c(1+4|\lceil n/2 \rceil |^3) t} (2|\lceil n/2 \rceil |^2)^{(1+4\lceil n/2 \rceil \varepsilon +2\sum ^n_{i=1}|\delta _i|)} [ 1 + \frac{1}{c^{2\varepsilon }(1-4\varepsilon )} ]^{ ( \lceil \nicefrac {n}{2} \rceil - \nicefrac {1}{2} ) } } \\  &\qquad \cdot \bigg [ \frac{ ( 1 - e^{ -ct } ) }{ 4|\lceil \nicefrac {n}{2}\rceil |^3c^{(2+4\lceil \nicefrac {n}{2}\rceil \varepsilon -2\sum ^n_{i=1}\delta _i)} } \sum _{ j, k = 1 }^\infty \frac{1}{ (j^2+k^2) } \bigg ]^{\nicefrac {1}{2}} = \infty . \end{aligned} \end{aligned}$$

We have thus established the fundamental step to prove item (v). Indeed, our choice of the vectors $v^{ k, r }_{ n,N } \in H$, $N,n \in {\mathbb {N}}$, $k \in {\mathbb {N}}_0$, $r \in {\mathbb {R}}$, in (64) and the condition $\sum ^n_{i=1} \delta _i > \frac{1}{2}$ have allowed us to establish (105). The infinite sum $\sum _{ j, k = 1 }^N \frac{1}{ (j^2+k^2) }$ appears on the right-hand side of (105), thus making the lower bound of the left-hand side of (106) infinite. Next we establish (112) below. To this end we note that (66) and (71) ensure that for all $n \in {\mathbb {N}}$, $\delta _1,\delta _2,\ldots ,\delta _n \in {\mathbb {R}}$, $\varepsilon \in (0,\nicefrac {1}{4})$, $m\in {\mathbb {N}}_0\cap [\frac{1}{4\varepsilon }-1,\infty )$ it holds that

107 $$\begin{aligned}&\sup _{N\in {\mathbb {N}}} \left[ \prod ^n_{i=1} \Vert \theta ^{n+1}_{i+1}(\textbf{u}^{\varepsilon ,m,(\delta _1,\delta _2,\ldots ,\delta _n)}_{n,N}) \Vert _{H_{-\delta _i}} \right] \nonumber \\  &\quad = \sup _{N\in {\mathbb {N}}} \bigg ( N^m \, \Vert v^{\lceil \nicefrac {n}{2} \rceil ,\delta _n-(\nicefrac {1}{2})-\varepsilon }_{\lceil \nicefrac {n}{2} \rceil N^m,N} \Vert _{H_{-\delta _n}} \prod ^{n-1}_{i=1} \Vert v^{\lceil \nicefrac {i}{2} \rceil ,\delta _i-\varepsilon }_{\lceil \nicefrac {n}{2} \rceil N^m,N} \Vert _{H_{-\delta _i}} \bigg )\nonumber \\  &\quad \le \bigg [ \sup _{N\in {\mathbb {N}}} \Big ( N^m \, \Vert v^{\lceil \nicefrac {n}{2} \rceil ,\delta _n-(\nicefrac {1}{2})-\varepsilon }_{\lceil \nicefrac {n}{2} \rceil N^m,N} \Vert _{H_{-\delta _n}} \Big )\bigg ] \, \prod ^{n-1}_{i=1} \bigg [ \sup _{N\in {\mathbb {N}}} \Vert v^{\lceil \nicefrac {i}{2} \rceil ,\delta _i-\varepsilon }_{\lceil \nicefrac {n}{2} \rceil N^m,N} \Vert _{H_{-\delta _i}} \bigg ]\nonumber \\  &\quad = \bigg [ \sup _{N\in {\mathbb {N}}} \Big ( N^{2m} \, \Vert v^{\lceil \nicefrac {n}{2} \rceil ,\delta _n-(\nicefrac {1}{2})-\varepsilon }_{\lceil \nicefrac {n}{2} \rceil N^m,N} \Vert ^2_{H_{-\delta _n}} \Big ) \bigg ]^{\nicefrac {1}{2}} \, \prod ^{n-1}_{i=1} \bigg [ \sup _{N\in {\mathbb {N}}} \Vert v^{\lceil \nicefrac {i}{2} \rceil ,\delta _i-\varepsilon }_{\lceil \nicefrac {n}{2} \rceil N^m,N} \Vert ^2_{H_{-\delta _i}} \bigg ]^{\nicefrac {1}{2}} . \end{aligned}$$

Furthermore, observe that (64) shows that for all $n\in {\mathbb {N}}$, $m\in {\mathbb {N}}_0$, $\delta \in {\mathbb {R}}$, $\varepsilon \in (0,\infty )$ it holds that

108 $$\begin{aligned} \begin{aligned}&\sup _{N\in {\mathbb {N}}} \Big ( N^{2m} \, \Vert v^{n,\delta -(\nicefrac {1}{2})-\varepsilon }_{nN^m,N}\Vert ^2_{H_{-\delta }} \Big ) = \sup _{N\in {\mathbb {N}}} \Big ( N^{2m} \, \Vert v^{n,-(\nicefrac {1}{2})-\varepsilon }_{nN^m,N}\Vert ^2_H \Big ) \\  &\quad = \sup _{N\in {\mathbb {N}}} \bigg ( N^{2m} \, \bigg \Vert {\textstyle \sum \limits ^N_{j=1}} \big [ (-A)^{-(\nicefrac {1}{2})-\varepsilon } e_{n+jnN^m} \big ] \bigg \Vert ^2_H \bigg ) \\  &\quad = \sup _{N\in {\mathbb {N}}} \left( \frac{N^{2m}}{ c^{(1+2\varepsilon )} } \Bigg [ \sum ^N_{j=1} \frac{1}{ (n+jnN^m)^{(2+4\varepsilon )} } \Bigg ] \right) \le \sup _{N\in {\mathbb {N}}} \Bigg ( \frac{N^{2m}}{ c^{(1+2\varepsilon )} } \Bigg [ \sum ^\infty _{j=1} \frac{1}{ (jN^m)^{(2+4\varepsilon )} } \Bigg ] \Bigg ) \\  &\quad = \sup _{N\in {\mathbb {N}}} \Bigg ( \frac{1}{ N^{4m\varepsilon } \, c^{(1+2\varepsilon )} } \Bigg [ \sum ^\infty _{j=1} \frac{1}{ j^{(2+4\varepsilon )} } \Bigg ] \Bigg ) = \frac{1}{ c^{(1+2\varepsilon )} } \Bigg [ \sum ^\infty _{j=1} \frac{1}{ j^{(2+4\varepsilon )} } \Bigg ] < \infty . \end{aligned} \end{aligned}$$

In addition, note that (64) and (103) imply that for all $n\in {\mathbb {N}}$, $i\in \{1,2,\ldots ,n\}$, $\delta \in {\mathbb {R}}$, $\varepsilon \in (0,\frac{1}{4})$, $m\in {\mathbb {N}}_0\cap [\frac{1}{4\varepsilon }-1,\infty )$ it holds that

109 $$\begin{aligned} \begin{aligned}&\sup _{N\in {\mathbb {N}}} \Vert v^{i,\delta -\varepsilon }_{nN^m,N}\Vert ^2_{H_{-\delta }} = \sup _{N\in {\mathbb {N}}} \Vert v^{i,-\varepsilon }_{nN^m,N}\Vert ^2_H = \sup _{N\in {\mathbb {N}}} \Vert Pv^{i,-\varepsilon }_{nN^m,N}\Vert ^2_H \le \frac{1}{c^{2\varepsilon }(1-4\varepsilon )} < \infty . \end{aligned}\nonumber \\ \end{aligned}$$

Combining (107) with (108) and (109) yields that for all $n \in {\mathbb {N}}$, $\delta _1,\delta _2,\ldots ,\delta _n \in {\mathbb {R}}$, $\varepsilon \in (0,\nicefrac {1}{4})$, $m\in {\mathbb {N}}_0\cap [\frac{1}{4\varepsilon }-1,\infty )$ it holds that

110 $$\begin{aligned} \begin{aligned}&\sup _{N\in {\mathbb {N}}} \left[ \prod ^n_{i=1} \Vert \theta ^{n+1}_{i+1}(\textbf{u}^{\varepsilon ,m,(\delta _1,\delta _2,\ldots ,\delta _n)}_{n,N}) \Vert _{H_{-\delta _i}} \right] < \infty . \end{aligned} \end{aligned}$$

Moreover, note that for all $N,n \in {\mathbb {N}}$, $k \in {\mathbb {N}}_0$, $r \in {\mathbb {R}}$ it holds that $v^{k,r}_{n,N} \in \operatorname {span}(\{e_m:m\in {\mathbb {N}}\}) {\setminus } \{0\}$. This and the fact that $\operatorname {span}(\{e_n:n\in {\mathbb {N}}\}) \subseteq \cap _{r\in {\mathbb {R}}} H_r$ ensure that for all $N,n\in {\mathbb {N}}$, $m \in {\mathbb {N}}_0$, $\varepsilon \in {\mathbb {R}}$, $\varvec{\delta } \in {\mathbb {R}}^n$ it holds that

111 $$\begin{aligned} \textbf{u}^{\varepsilon ,m,\varvec{\delta }}_{n,N} \in \big ( (\cap _{r\in {\mathbb {R}}} H_r) \setminus \{0\} \big )^{n+1}. \end{aligned}$$

Combining this with (110) assures that for all $n \in {\mathbb {N}}$, $\varvec{\delta } \in {\mathbb {R}}^n$, $\varepsilon \in (0,\frac{1}{4})$, $m\in {\mathbb {N}}_0\cap [\frac{1}{4\varepsilon }-1,\infty )$ it holds that

112 $$\begin{aligned} \inf _{N\in {\mathbb {N}}} \left[ \frac{1}{ \prod ^n_{i=1} \Vert \theta ^{n+1}_{i+1}(\textbf{u}^{\varepsilon ,m,\varvec{\delta }}_{n,N}) \Vert _{H_{-\delta _i}} } \right] = \frac{1}{ \big [ \sup _{N\in {\mathbb {N}}} \big ( \prod ^n_{i=1} \Vert \theta ^{n+1}_{i+1}(\textbf{u}^{\varepsilon ,m,\varvec{\delta }}_{n,N}) \Vert _{H_{-\delta _i}} \big ) \big ] } \in (0,\infty ). \end{aligned}$$

This, (106), and (111) show that for all $n \in {\mathbb {N}}$, $q \in [0,\infty )$, $\varvec{\delta }=(\delta _1, \delta _2, \ldots , \delta _n) \in {\mathbb {R}}^n$, $\varepsilon \in (0,\frac{\lceil \nicefrac {n}{2}\rceil }{2})$, $m\in {\mathbb {N}}_0\cap [\frac{\lceil \nicefrac {n}{2}\rceil }{2\varepsilon }-1,\infty )$, $t \in (0,T]$ with $\sum ^n_{i=1} \delta _i \ge \frac{1}{2} + \varepsilon$ it holds that

113 $$\begin{aligned} \begin{aligned}&\sup _{ \textbf{u}=(u_0,u_1,\ldots ,u_n) \in ({(\cap _{r\in {\mathbb {R}}}H_r)}\setminus \{0\})^{n+1} } \left[ \frac{ \big ({\mathbb {E}}\big [\Vert X^{ n,\textbf{u} }_t \Vert ^2_{H_{-q}}\big ]\big )^{\nicefrac {1}{2}} }{ \prod ^n_{ i=1 } \Vert u_i\Vert _{H_{-\delta _i}} } \right] \\  &\quad = \sup _{ \textbf{u}=(u_0,u_1,\ldots ,u_n) \in ({(\cap _{r\in {\mathbb {R}}}H_r)}\setminus \{0\})^{n+1} } \left[ \frac{ \big ({\mathbb {E}}\big [\Vert X^{ n,\textbf{u} }_t \Vert ^2_{H_{-q}}\big ]\big )^{\nicefrac {1}{2}} }{ \prod ^n_{ i=1 } \Vert \theta ^{n+1}_{i+1}(\textbf{u})\Vert _{H_{-\delta _i}} } \right] \\  &\quad \ge \sup _{ N \in {\mathbb {N}}} \left[ \frac{ \big ({\mathbb {E}}\big [\Vert X^{ n,\textbf{u}^{\nicefrac {\varepsilon }{(2\lceil \nicefrac {n}{2}\rceil )},m,\varvec{\delta }}_{n,N} }_t \Vert ^2_{H_{-q}}\big ]\big )^{\nicefrac {1}{2}} }{ \prod ^n_{i=1} \Vert \theta ^{n+1}_{i+1}(\textbf{u}^{\nicefrac {\varepsilon }{(2\lceil \nicefrac {n}{2}\rceil )},m,\varvec{\delta }}_{n,N}) \Vert _{H_{-\delta _i}} } \right] \\  &\quad \ge \left[ \inf _{N\in {\mathbb {N}}} \frac{1}{ \prod ^n_{i=1} \Vert \theta ^{n+1}_{i+1}(\textbf{u}^{\nicefrac {\varepsilon }{(2\lceil \nicefrac {n}{2}\rceil )},m,\varvec{\delta }}_{n,N}) \Vert _{H_{-\delta _i}} } \right] \left[ \sup _{ N \in {\mathbb {N}}} \big ({\mathbb {E}}\big [\Vert X^{ n,\textbf{u}^{\nicefrac {\varepsilon }{(2\lceil \nicefrac {n}{2}\rceil )},m,\varvec{\delta }}_{n,N} }_t \Vert ^2_{H_{-q}}\big ]\big )^{\nicefrac {1}{2}} \right] =\infty . \end{aligned} \end{aligned}$$

This and Hölder’s inequality establish item (v). The proof of Theorem 2.1 is thus completed.
